# Processes that drive the population structuring of *Jenynsia lineata* (Cyprinidontiformes, Anablepidae) in the La Plata Basin

**DOI:** 10.1002/ece3.7427

**Published:** 2021-05-08

**Authors:** Yanina F. Briñoccoli, Luiz Jardim de Queiroz, Sergio Bogan, Ariel Paracampo, Paula E. Posadas, Gustavo M. Somoza, Juan I. Montoya‐Burgos, Yamila P. Cardoso

**Affiliations:** ^1^ Laboratorio de Ictiofisiología y Acuicultura Instituto Tecnológico Chascomús (CONICET‐UNSAM) Chascomús Argentina; ^2^ Department of Genetics and Evolution University of Geneva Geneva Switzerland; ^3^ Fundación de Historia Natural “Félix de Azara” Departamento de Ciencias Naturales y Antropología Universidad Maimónides Ciudad Autónoma de Buenos Aires Argentina; ^4^ Instituto de Limnología Dr. Raúl A. Ringuelet CONICET‐CCT La Plata‐UNLP Buenos Aires Argentina; ^5^ CONICET Laboratorio de Sistemática y Biología Evolutiva (LASBE) Facultad de Ciencias Naturales y Museo Universidad Nacional de La Plata Buenos Aires Argentina

**Keywords:** basin fragmentation, COI, genetic structure, intraspecific variation, isolation

## Abstract

1. The distribution of genetic diversity across a species distribution range is rarely homogeneous, as the genetic structure among populations is related to the degree of isolation among them, such as isolation by distance, isolation by barrier, and isolation by environment.
2. *Jenynsia lineata* is a small viviparous fish that inhabits a wide range of habitats in South America. To decipher the isolation processes that drive population structuring in *J. lineata,* we analyzed 221 sequences of the mitochondrial *cytochrome c oxidase I gene* (COI), from 19 localities. Then, we examined the influence of the three most common types of isolation in order to explain the genetic variation found in this species.
3. Our results revealed a marked structuration, with three groups: (a) La Plata/Desaguadero Rivers (sampling sites across Argentina, Uruguay, and Southern Brazil), (b) Central Argentina, and (c) Northern Argentina. A distance‐based redundancy analysis, including the explanatory variables geographical distances, altitude, latitude, and basin, was able to explain up to 65% of the genetic structure. A variance partitioning analysis showed that the two most important variables underlying the structuration in *J. lineata* were altitude (isolation by environment) and type of basin (isolation by barrier).
4. Our results show that in this species, the processes of population diversification are complex and are not limited to a single mechanism. The processes that play a prominent role in this study could explain the high rate of diversity that characterizes freshwater fish species. And these processes in turn are the basis for possible speciation events.

## Graphical abstract

The processes of population diversification are complex and are not limited to a single mechanism. Population structuring may lead to population reproductive isolation and ultimately to speciation.

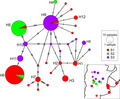

## 1 INTRODUCTION

The distribution of genetic diversity across a species distribution range is rarely homogeneous since there is a genetic structure related to the degree of isolation that may exist among groups of individuals. The population structure can be expressed as different patterns. The three most common patterns of structuration are as follows: (a) by geographical distance (isolation by distance, IBD (Wright, 1943)); (b) by environmental heterogeneity (isolation by environment, IBE (Wang & Bradburd, 2014)); and (c) by physical barriers (isolation by barrier, IBB (Rahel, 2007)).

Regarding the geographic distance, the genetic similarity among populations tends to decay when geographical distance increases (Wright, 1943), which leads to the IBD pattern. Such a pattern is notable in organisms with intermediate or limited dispersal ability (Peterson & Denno, 1998; Shurin et al., 2009) such as *Serrasalmus rhombeus* (Hubert & Renno, 2006) and *Prochilodus nigricans* (Machado et al., 2017), both showing migrations up to 100 km. On the other hand, organisms with high capacity of dispersal tend to show a weaker or no IBD pattern, populations being more genetically homogeneous. In rivers, this geographical distance between study points in a riverscape is poorly estimated when calculated based on the geographical coordinates of the localities (i.e., Euclidean distance) because the distance separating two localities is generally not a straight line. This may not be ecologically representative because it fails to take into account the spatial configuration, connectivity, directionality, and relative position of sites in a river network (Rouquette et al., 2013). When environments are complex and heterogeneous, aside from the role of dispersal ability, populations may face strong migration resistance due to the patchiness of their preferred habitat. This is called environmental fragmentation (Kershenbaum et al., 2014). Here, the heterogeneity of substrate, salinity, or water temperature (Gonzalez et al., 2016; McCairns & Bernatchez, 2008) can drive to genetic structuring, generating patterns of IBE (Wang & Bradburd, 2014). In the particular situation of IBE, populations from similar environments, independently of the geographic distance, should show the highest rate of gene flow (Sexton et al., 2014). Or, as already been seen in coral reefs, environmental gradients in conjunction with geographic distance can influence gene flow patterns (Nanninga et al., 2014). This pattern of isolation, IBE, may arise, for instance, as a result of natural or sexual selection against immigrants or according to the reduction in their fitness caused by population hybridization (Wang & Bradburd, 2014). As such, ecological speciation can be one of the final products of this evolutionary process (Nosil, 2012).

Finally, topographic barriers and landscape breaks such as waterfalls, dams, or basin fragmentation due to climate change (Dias et al., 2012; Jardim de Queiroz et al., 2017; Rahel, 2007; Winemiller et al., 2008) are known to have an impact on the population isolation process. In South America, several works have shown how these climatic fluctuations affect the different bodies of water. Mar Chiquita, an extensive saline lake located in central Argentina, is a sensitive climatic marker of rainfall fluctuations, where historical data and current measurements have shown that during the dry historical intervals, the lake surface was reduced by ~2,000 km^2^, while in the periods with a positive hydrological balance, the lake has covered an area ~6,000 km^2^ greater than nowadays (Piovano et al., 2006; Troin et al., 2010). Other examples such as in the Salado–Juramento rivers (Thalmeier et al., 2021) and in the Pilcomayo River (Smolders et al., 2002) also demonstrate how climatic fluctuations have impacted river continuity and water flow. As a resulting pattern, the IBB is very often associated with a very abrupt structuration with geographic space since populations very close to each other, yet separated by a barrier, will have very low gene flow; this results in a very high genetic dissimilarity. This phenomenon of basin fragmentation isolates the aquatic organisms that inhabit these rivers generating genetic diversification (Berry et al., 2019). If the isolation time is long enough, it will cause population genetic differentiation within the species that have been fragmented. But if the isolation is even greater, it may eventually produce new species via allopatric speciation.

In this study, we test the basin fragmentation–reconnection hypothesis that may lead to IBB. This hypothesis states that during dry geological periods, the level of rainfall is low, generally discontinuous and concentrated in the mountains due to the convection effect. During these dry periods, some of the rivers in the upper regions of the South American continent may have suffered a significant decrease in their water levels, preventing them from reaching the rest of the system's water network, and reducing their channel to savannas or arid areas (Albert & Reis, 2011). This is one mechanism by which a basin can fragment into various endorheic (or arheic) systems. Other mechanisms that may cause basin fragmentation or reconnection are the tectonic and volcanic processes which can modify the regional topography, and which, together with the climate, affect weathering, erosion, sediment transport thus may modulate river connectivity (Gawthorpe & Leeder, 2000; Scholz & Rosendahl, 1988). The organisms that inhabit these endorheic basins are isolated from the main system (exorheic). On the contrary, during wet geological periods, the elevated amount of rain increases the water flowing into the rivers (Berry et al., 2019; Masiokas et al., 2019). With an increased water flow, river systems can achieve greater distribution. During these geological periods, rivers that were disconnected could reconnect to the main system forming again a large unified system. Today, the climate in northeast and central Argentina is temperate (with an average annual temperature of 16.2°C) with rainy seasons between October and April (about 900 mm per year) (Díaz Zorita et al., 1998). This climate corresponds to dry geological periods (Piovano et al., 2006; Wang et al., 2018). However, the climate of this region was not constant throughout history. Several studies have shown that the intertropical rainfall regimes have changed during the Miocene (Rea, 1994), Pliocene (Gladstone et al., 2007), and Pleistocene (Broccoli et al., 2006), strongly affecting the climate in the South American continent.

The Neotropical genus *Jenynsia* Günther, 1866 is a group of fish comprising 15 species. They are small viviparous fishes that inhabit a wide range of habitats, spanning from mountain rivers in the Andes to floodplains and large rivers as Río de la Plata estuary (Aguilera & Mirande, 2005; Aguilera et al., 2013; Amorim & Costa, 2019; Calviño & Alonso, 2016; Frota et al., 2019, 2020). Although the genus has wide distribution, found from Rio de Janeiro in Brazil, to the province of Rio Negro in Argentina, most species show a very geographically limited distribution, often inhabiting small exclusive drainages (Ghedotti & Weitzman, 1996; Aguilera & Mirande, 2005; Lucinda et al., 2006; Aguilera et al., 2013; 2019). An exception to this distribution pattern is *Jenynsia lineata* (Jenyns, 1842) which inhabits both uplands, up to 2,300 MASL (Meters Above Sea Level), and lowlands across Argentina, Brazil, and Uruguay (Aguilera & Mirande, 2005; Amorim, 2018; Frota et al., 2019; Ghedotti & Weitzman, 1996). According to Amorim & Costa, 2019, this species, originally from freshwaters, invaded brackish waters in the Early Pleistocene (1.1 Ma, 95% HPD 3.6–0.2 Ma) and the range of distribution of the species was expanded.

The distribution range of *Jenynsia lineata* covers most of the La Plata Basin. This hydrographic basin is the second largest in the world and occupies important territories belonging to Argentina, Bolivia, Brazil, Uruguay, and Paraguay. In its large extension, different natural aquatic environments can be found, ranging from typical freshwater to those where it mixes with seawater, then forming an estuary ecosystem, as it happens in the mouth of the Rio de la Plata. In this context, the clear predominance of plains stands out, which makes it very susceptible to events of fragmentation–reconnection of basins during climatic changes in wet and dry periods. In fact, between the Andes and the plain of the La Plata Basin there are several rivers that have their flow interrupted, which disconnects them from the rest of the basin. These interrupted rivers end either into lakes or lagoons with no outlets (called endorheic rivers), or they infiltrate the ground until they disappear (arheic rivers). Such endorheic or arheic rivers are frequent from the northwestern Argentinian Pampa up to southern Bolivia and they are the result of dry periods (Ciccioli et al., 2018; Wirrmann & Mourguiart, 1995). On the contrary, all rivers that reach the main drainage net are called exorheic rivers.

The wide distribution of *Jenynsia lineata* makes it an interesting model to address the role of basin fragmentation–reconnection process on diversification as an IBB pattern; it constitutes a solid case study because this species is present in both endorheic and exorheic basins. Moreover, no studies have been conducted in fish to elucidate the presence of genetic structuring due to this kind of fragmentation. In this study, we test for the three main patterns of population structure (IBD, IBE, and IBB) and examine the possible underlying processes. Particularly for IBB, we test whether the events of fragmentation–reconnection of basins have played a role in the population structuration of *J. lineata*.

## 2 MATERIAL AND METHODS

### 2.1 Sampling and sequence data

In Argentina, fish were collected with the permission of the local authorities. The study was approved by the National Council of Scientific and Technical Research of Argentina (exp. 7879/14) and it is a requirement of this institution to follow the guidelines of its "Comité de Etica" (https://www.conicet.gov.ar/wp‐content/uploads/OCR‐RD‐20050701‐1047.pdf) and its biological sampling guide (https://proyectosinv.conicet.gov.ar/solicitud‐colecta‐cientifica/). Also, fish handling during sampling was performed following guidelines of the UFAW Handbook on the Care and Management of Laboratory Animals (http://www.ufaw.org.uk). Fish were anesthetized and killed using water containing a lethal dose of eugenol (clove oil).

**FIGURE 1:**
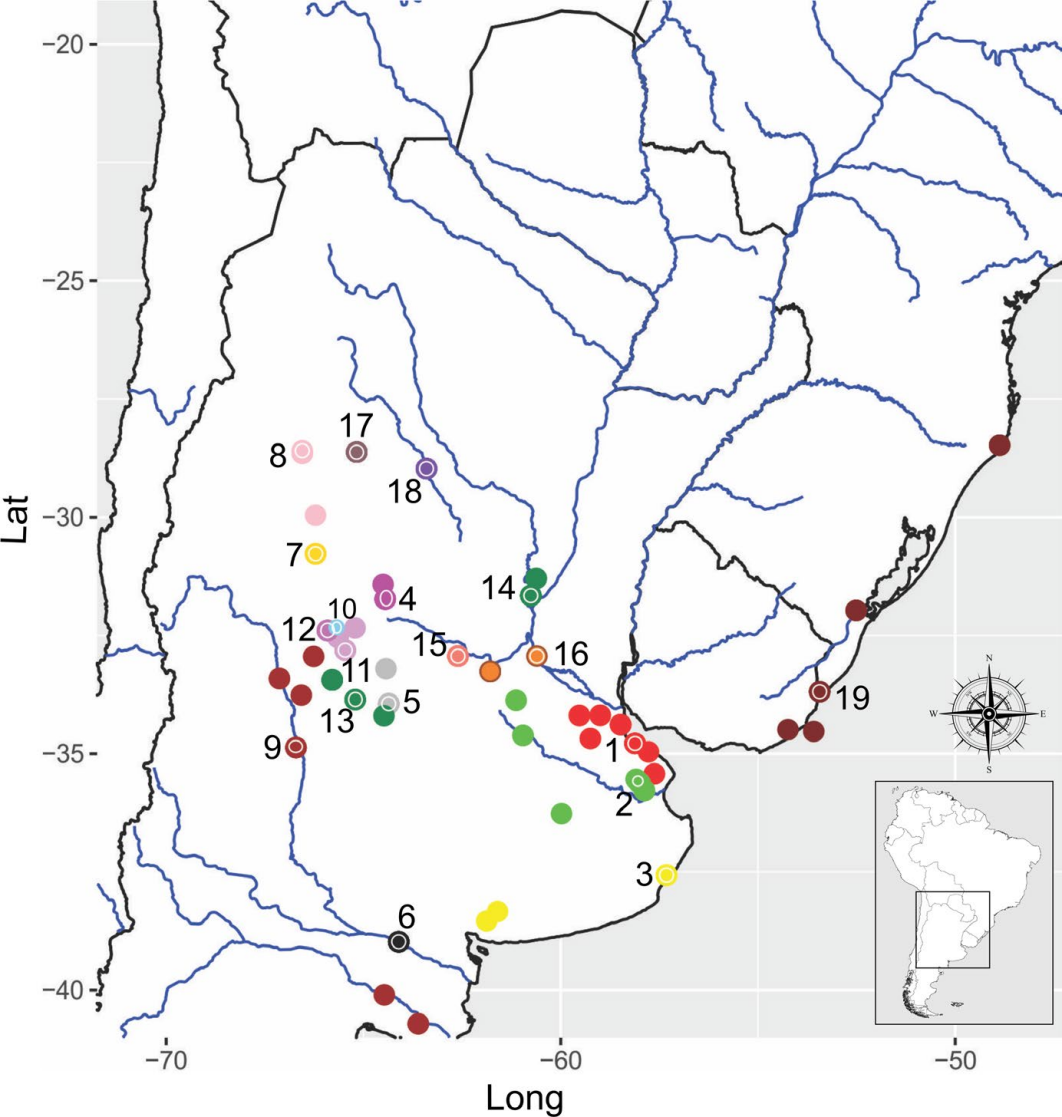
Study area, including the 57 sampling areas colored by locations (19 localities). The points with inner white circle indicate the chosen final locations

Samples of *Jenynsia lineata* were collected in 57 sites distributed across Argentina, Uruguay, and Brazil (Figure 1) covering the species distribution. The samples were grouped in 19 localities according to the distance among the sites and the basin to which they belonged (Table 1; Table S1). The sampling sites were classified in different categories: according to the hydrographic system, the type of basin, the altitude, and the latitude (explained in the following analyzes).

**TABLE 1:** Sampling localities and genetic diversity indices based on the COI gene for *Jenynsia lineata*

System/Basin	Location	Long	Lat	n	Altitude group	h	s	pi	Tajima's D	Gene diversity
La Plata EX	**1**	−**58.11719**	−**34.782692**	**22**	1	**5**	**9**	**1.472**	−**1.353**	**1.0000** *
	**2**	−**57.997858**	−**35.617486**	**16**	1	**4**	**6**	**1.833**	**0.047**	**1.0000** *
	**3**	−**57.317256**	−**37.566633**	**12**	1	**2**	**5**	**1.621**	−**0.077**	**1.0000** *
	**5**	−**64.359861**	−**33.94333**	**15**	**2**	**1**	**1**	**0.133**	−**1.159**	**1.0000** *
	**14**	−**60.757444**	−**31.657111**	**14**	**1**	**3**	**3**	**1.033**	**0.291**	**1.0000** *
	**15**	−**61.785694**	−**33.26**	**7**	**2**	**1**	**0**	**0**	**0**	**1.0000**
	**16**	−**62.603713**	−**32.93637**	**13**	**1**	**2**	**2**	**0.923**	**1.214**	**1.0000** *
Mar Chiquita EX	**4**	−**64.44881**	−**31.729583**	**10**	**4**	**2**	**2**	**0.4**	−**1.401** *	**1.0000** *
	**18**	−**63.462222**	−**29.3756**	**12**	**2**	**1**	**0**	**0**	**0** *	**1.0000** *
Río Colorado‐Negro EX	**6**	−**64.106117**	−**38.976575**	**9**	1	**2**	**2**	**1**	**1.235**	**1.0000** *
	**9**	−**66.709994**	−**34.86956**	**13**	3	**3**	**9**	**2.462**	−**0.596**	**1.0000** *
Salar de Pipanaco EN	**7**	−**66.21297**	−**30.772233**	**6**	**4**	**4**	**3**	**1.267**	−**0.185**	**1.0000**
	**8**	−**66.54917**	−**28.6494**	**5**	4	**3**	**8**	**4.4**	**1.027**	**1.0000**
Sierras de San Luis EN	**10**	−**65.721722**	−**32.415639**	**9**	**5**	**8**	**17**	**5.389**	−**0.674**	**1.0000** *
	**11**	−**65.46175**	−**32.810472**	**14**	**5**	**2**	**6**	**2.967**	**2.055**	**1.0000** *
	**12**	−**65.913361**	−**32.396361**	**6**	**4**	**2**	**8**	**4.267**	**1.284**	**1.0000**
Río Quinto EN	**13**	−**65.79215**	−**33.440733**	**12**	**4**	**1**	**9**	**1.5**	−**2.016** *	**1.0000** *
Salar Ambargasta EN	**17**	−**65.170667**	−**28.62328**	**14**	**3**	**2**	**1**	**0.143**	−**1.155**	**1.0000** *
Este Uruguay EX	**19**	−**53.43638**	−**33.689444**	**12**	**1**	**5**	**8**	**2.833**	**0.278**	**1.0000** *
	**TOTAL**			**221**	**4**	**32**	**5.1**	**1.728**	*	

Note

Long = longitude; Lat = latitude; n = sample size; h = number of haplotypes; s = *N* of observed sites with substitutions; pi = mean *N* of pairwise differences.

The sublocalities and geographical coordinates in bold were those chosen to represent the locality.

* *p* significant.

Fish were caught with seine nets or trawl nets. The specimens collected were identified using the original descriptions and updated taxonomical literature (Amorim, 2018). Morphological vouchers were deposited in the fish collections of the Fundación de Historia Natural “Félix de Azara,” Buenos Aires (CFA‐IC). Tissue samples were stored in 98% ethanol, and DNA was extracted using the commercial peqGOLD Tissue DNA Mini Kit (PEQLAB Biotechnologie GmbH, DEU). A total of 244 sequences of *Jenynsia* were analyzed. From the 221 sequences belonging to *J. lineata* (Table 1; Table S1), 180 sequences are new for this study while the remaining 41 were obtained from GenBank (Table S2). The remaining 23 sequences belong to four species of closely related genera (Amorim & Costa, 2019) were added as out‐groups (*Anableps microlepis* Müller & Troschel 1844*, Oxyzygonectes dovii* (Günther 1866)*, Gambusia holbrooki* Girard 1859 and *Phalloceros caudimaculatus* (Hensel 1868). A 598‐base pair (bp) of the mitochondrial *cytochrome c oxidase I gene* (COI) was amplified by polymerase chain reaction (PCR) from each fish sample. The following primers were used for COI amplification: SILCOI‐D and SILCOI‐R (Jardim de Queiroz et al., 2020). The amplifications were performed in a final volume of 25 µl containing 1X Green GoTaq Reaction Buffer, 0.2 mM dNTP mix, 0.5 µM of each primer, 1.25 U GoTaq DNA polymerase (Promega, Madison, WI, USA), and 50–100 ng of DNA template. The amplification protocol consisted of 95°C for 2 min; 35 cycles of 94°C for 30 s, 52–56°C for 30 s, and 72°C for 1 min; and a final extension of 72°C for 10 min. PCR products were visualized in a 1% agarose gel. Purification and sequencing, in one direction, were performed by the company Macrogen, Inc. (Seoul, South Korea). The sequences were aligned using BioEdit 7.1.3.0 (Hall, 1999).

### 2.2 Phylogenetic reconstruction and haplotype network

To test the monophyly of the *Jenynsia lineata* samples, a phylogenetic reconstruction was performed with maximum likelihood (ML) using MEGA 7.0.26 (Kumar et al., 2016). The reliability values of the nodes were obtained by 1,000 bootstrap replicas (Felsenstein, 1985). In the ML analysis, the optimal nucleotide substitution model (HKY + G) was selected according to the Bayesian information criterion (BIC) by JModelTest 2.1.10 (Darriba et al., 2015).

### 2.3 Population genetic analyses

Descriptive statistics and genetic structure analyses were based on the partial COI gene. Standard diversity indices (number of haplotypes, number of variable sites, gene diversity, and nucleotide diversity (π)) and Tajima's D were calculated in Arlequin 3.5.2.2 (Excoffier & Lischer, 2010). Genetic differentiation between localities was measured by calculating pairwise‐FST values with the TN93 correction, which was the best substitution model available that fit the data in Arlequin 3.5.2.2. The statistical significance of FST was assessed using 10,000 permutations of individuals among the 19 localities.

To assess the role of geographical distance on genetic structuration, we used the Mantel test (Mantel, 1967). We built a matrix of genetic distance by localities and a matrix of geographical distance. However, the geographical distance between localities in a riverscape is poorly estimated when calculated based on the geographical coordinates of the localities (the distance separating two localities is generally not a straight line). Thus, geographical distances following the course of the rivers were calculated with Google Earth Pro each distance between pairs of localities was estimated in kilometers, with which a matrix was made. For the localities that were in endorheic rivers, the distances were calculated following a probable route of connection between them. On the other hand, for the localities present in Brazil and Uruguay, they were connected with the others through the connection of the sea with the Rio de La Plata. This matrix was used for the Mantel test.

To assess the population structure without imposing a priori groupings of localities, we performed several spatial analyses of molecular variance (SAMOVA) using SAMOVA 1.0 (Dupanloup et al., 2002). The significance of the fixation indices was tested with 10,000 permutations. We tested k‐values (number of groups) ranging from 2 to 10 and considered the combination of high FCT(i.e., proportion of variation among groups or the relative level of genetic variations among groups) without finding much improvement as an indicator of the best structure pattern. We first performed SAMOVA taking into account the geographical coordinates of the localities as points on a map. To take into consideration the real distance between the localities following the course of the rivers, we converted the matrix of geographical distance between pairs of localities united by the course of the rivers into a two‐dimensional metric multidimensional scale (MDS) transformation (Abdi, 2007). This is a mean of visualizing the level of similarity among data, in this case sampling points. The MDS analysis was performed using the R package vegan (Oksanen et al., 2007). Moreover, the values given by MDS, which represent the distances in two dimensions, were also used to run SAMOVA.

To test the different patterns of genetic structuration in *J. lineata*, we applied AMOVAs on the COI dataset using Arlequin 3.5.1.3. We performed several tests grouping the data by basin, hydrographic system, and altitude.

We also graphed the minimum spanning network of the COI haplotypes using PopART 1.7 (Leigh & Bryant, 2015). The network was colored based on the following classifications: hydrographic system, basin (these two, in search of an isolation by barrier pattern, explained in the next section), and altitude (in order to identify any isolation by environment pattern).

### 2.4 Distance‐based Redundancy Analysis—db‐RDA

We performed distance‐based redundancy analysis (db‐RDA) to unravel the variables explaining the genetic differentiation of *J. lineata* among localities. To do so, we adapted the db‐RDA script published by Jardim de Queiroz et al. (2017). We used the pairwise‐FST matrix as the response variable in the db‐RDA. To test the effect of regional and environmental variables in genetic distance, we used the following explanatory variables:

1. The geographical distance between each pair of sampling sites (to test for isolation by distance, IBD): For this analysis, we used the geographical distance calculated by considering the course of the rivers instead of using the Euclidean distance extracted from the geographical coordinates. To transform the matrix of geographical distance into vectors, we applied a Principal Coordinates of Neighbour Matrices (PCNM) by using the package PCNM (Legendre et al., 2013) and its function “PCNM,” following the methodology described in Borcard and Legendre (2002). In our analyses, the first six axes (out of a total of 10) were found to have positive eigenvalues and were kept for the db‐RDA.
2. Basin type (representing possible isolation by barrier, IBB): We incorporated a dummy variable in the model according to the type of basin of each sampling site: The sites were categorized either as “0” if exorheic basins—have connection with the main La Plata Basin or the sea—(localities: 1, 2, 3, 5, 6, 9, 14, 15, 16, 19, 20), or “1” if endorheic basins—without connection—(localities: 4, 7, 8, 10, 11, 12, 13, 17, 18).
3. Hydrographic system (representing possible IBB): The sampling sites were classified in 8 systems: (a) Mar Chiquita, (b) La Plata, (c) Río Quinto, (d) Salar Ambargasta, (e) Río Colorado‐Negro, (f) Salar de Pipanaco, (g) Sierras de San Luis, and (i) Este Uruguay; these were taken as factors since it was necessary to give them a numerical value for the analysis.
4. Altitude (representing isolation by environment, IBE): As *J. lineata* is present from lowlands to up to 2,300 MASL, we used altitude data inferred for each sampling site in our model. Values were taken from Google Earth Pro. However, contrary to the haplotype network reconstruction where we classify the localities into groups of altitudes, in which we used five categories of altitude (i.e., (a) group 1 from 0 to 99 MASL, (b) group 2 from 100 to 299 MASL, (c) group 3 from 300 to 499 MASL, (d) group 4 from 500 to 699 MASL, and (e) group 5 from 700 MASL), for the db‐RDA we used this variable as a continuous variable.
  Elevational gradients may be challenging for most species and can be considered as IBE for many reasons: (a) The number of species increases downstream with a marked difference in species' richness between upland and lowland areas (Bistoni & Hued, 2002). Studies of the changes resulting from the correlation of altitude with biodiversity have included a wide range of organisms, including vertebrates, invertebrates, and plants from many different geographic regions (Tobes et al., 2016). (b) The water flow is different between lowlands and uplands. In uplands, the headwaters of the rivers are fast flowing due to more pronounced slopes, which leads to a strong erosive power that affects the physicochemical properties of the water: high oxygen, low conductivity, and low levels of nutrients. (c) The area covered by a basin is larger in lowlands. (d) The physical and chemical conditions influence the distribution of fish in aquatic ecosystems (Buisson et al., 2008). In addition, physiological and morphological adaptations (hydrodynamic shapes), as well as low metabolic rates, are conditions necessary for fish survival (Beitingera et al., 2000; Taniguchi & Nakano, 2000; Winemiller et al., 2008).
5. Latitude (representing IBE): For *J. lineata*, each sampling site has a coordinate measured in decimals, both for latitude and longitude. For this analysis, we only took into account the list of latitudinal coordinates for each location.
  The gradients across latitudes in the La Plata Basin imply changes in community composition and climatic variation. Therefore, we added latitude in our model as a proxy for environmental heterogeneity. This variable was included in the model as decimals, measured according to the geographical coordinates of each site's south latitude. The latitudinal gradients of species richness for fishes generally corroborated the paradigm of latitudinal diversity gradient (LDG) (Willig et al., 2003), which encompasses the tendency of biological diversity to concentrate in tropical regions. This LGD is ultimately dependent on historical, geographic, biotic, abiotic, and stochastic forces (Schemske, 2002), which affect the geometry, internal structure, and location of species ranges in ecological or evolutionary time. Specifically, latitude is a surrogate for a number of primary environmental gradients (e.g., temperature, insolation, seasonality) that interact and are correlated to each other. With regard to the species' richness of fishes, it is considered that it increases with decreased latitude throughout the world for marine and freshwater taxa as well as for assemblages in lentic and lotic habitats (Barbour & Brown, 1974; Hof et al., 2008; Willig et al., 2003).

Before starting the analyses, we performed a Pearson test (for the quantitative) and a chi‐square (for the qualitative) variables to test for the independence of the variables. Then, to identify the variables that explain part of the genetic structure, we first ran a db‐RDA on the full model (including all investigated variables) using the function “capscale” of the package vegan (Oksanen et al., 2007). Then, we ran a db‐RDA on nested models to identify the best model based on Akaike information criterion (AIC). As db‐RDA does not provide information on the relative contribution of each variable of the model, we performed a variance partitioning analysis on the variables present in the best model to identify their relative contribution. For that, we used the function “varpart” of the package vegan in the R environment (Peres‐Neto et al., 2006).

## 3 RESULTS

### 3.1 Genetic structure, demography, and testing explanatory factors with AMOVA

**FIGURE 2:**
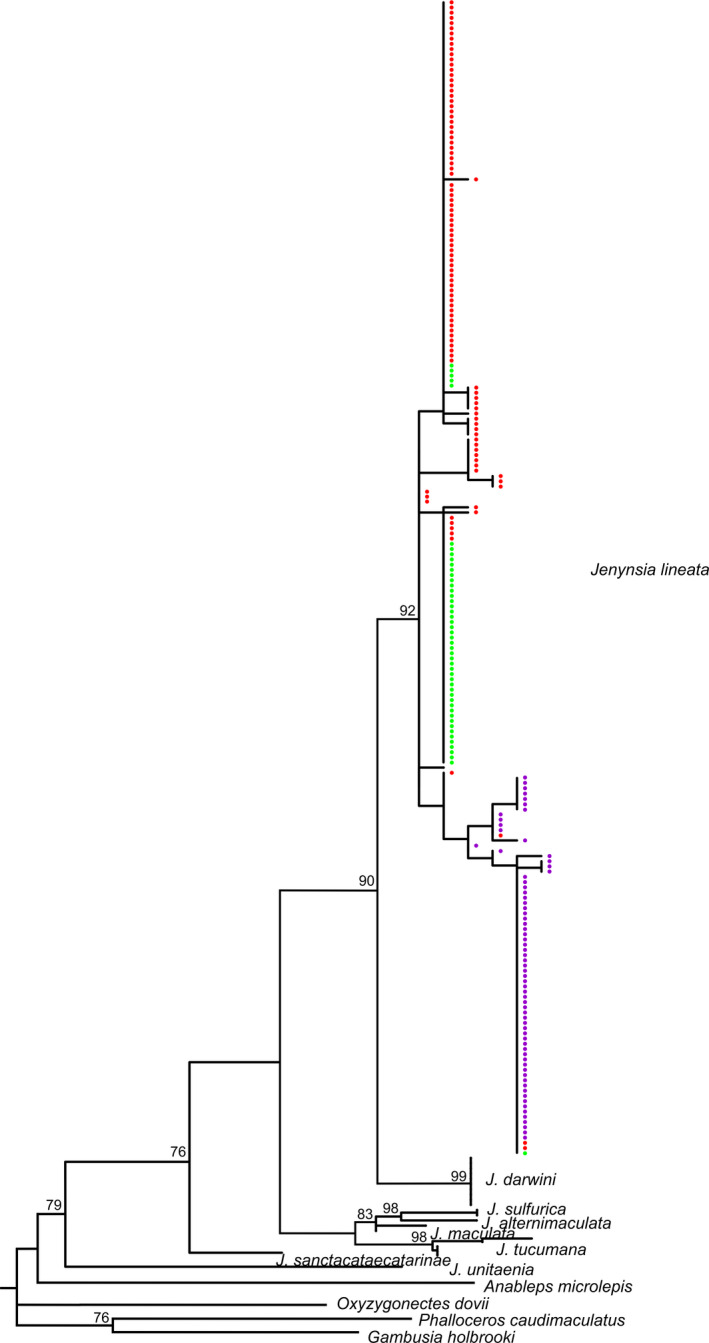
Maximum‐likelihood tree of *Jenynsia lineata* based on 598 nucleotides of the mitochondrial gene COI. *J. lineata* sequences are represented by colored dots to 3 groups given by SAMOVA. Bootstrap values are shown above the branches. Values below 70 are not shown

The monophyly of the *J. lineata* sequences used in this study was confirmed by our phylogenetic reconstruction, since all the sequences grouped together in a single clade with a support value of 92% (Figure 2).

**FIGURE 3:**
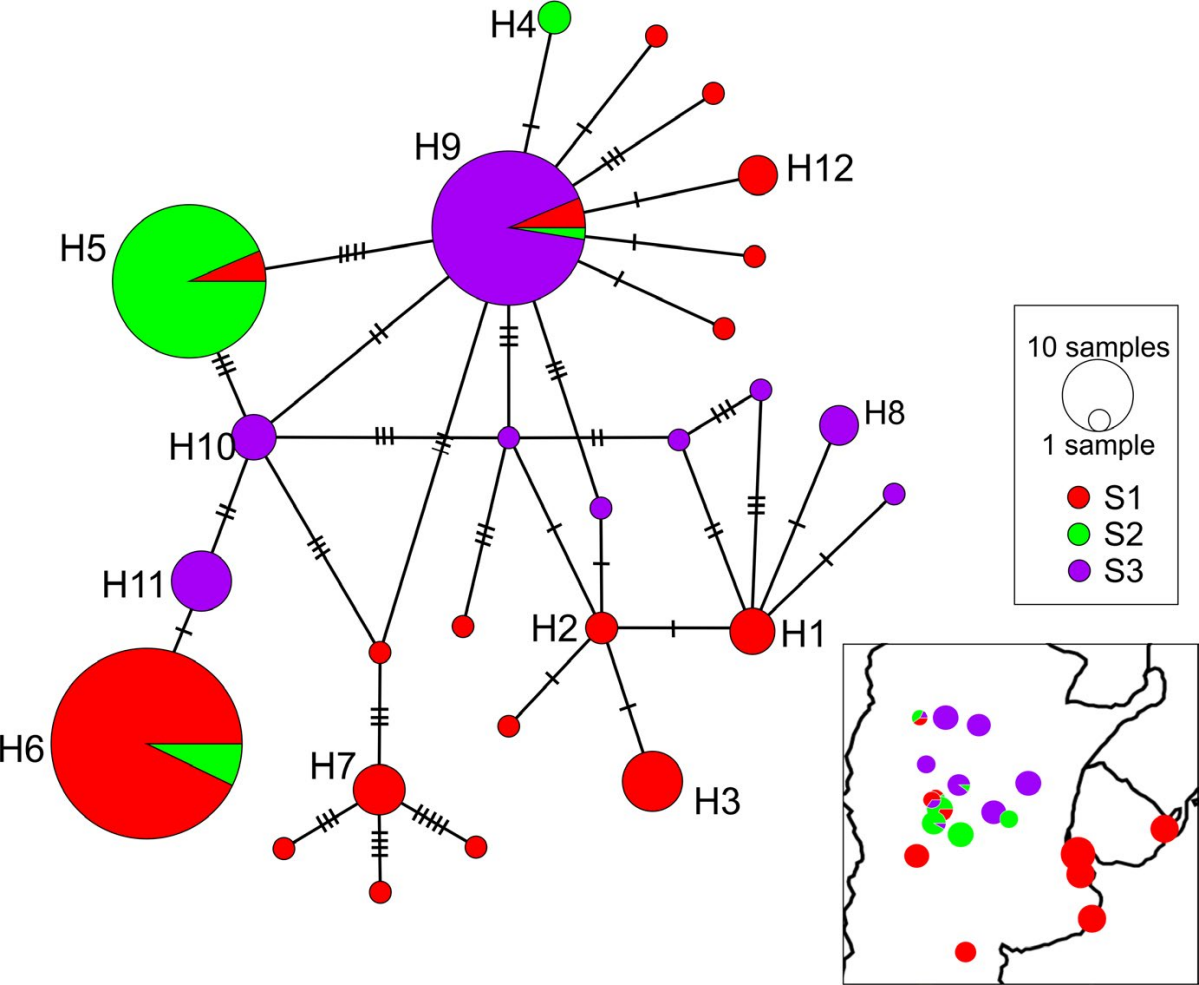
Haplotype network, colored by three SAMOVA groups and maps of the distribution range of each group

We identified 12 different haplotypes of COI (Table 1; Figure 3; Figure S1). The haplotype network shows the presence of three major haplogroups with shared haplotypes among populations. The haplotypes were found related in a complex network (Figure 3; Figure S1). Regarding the values of Tajima's D, the majority were not significant, but about half of the localities (10 out of 19) resulted in positive values.

The genetic differentiation among localities (pairwise‐FST values) indicated a low genetic differentiation in *J. lineata*, even between remote localities (Table S3). The highest FST values were closed to 1.7%.

We tested the correlation between both geographic matrices (i.e., connecting localities in a straight line and following the course of the rivers) with the genetic distance matrix. In both cases, the Mantel test results were not significant (*p *= 0.129 and 0.457, respectively).

Both SAMOVA with geographic distance following the course of the rivers (Table S4‐a) and based on the Euclidean distance (Table S4‐b) suggested that an organization into three groups or populations (*K* = 3, for both analyzes, the groups were made up of the same localities) best reflects the genetic structure of this species in the region. The FCT values did not increase substantially with an increasing number of groups (FCT = 0.744 in both analyses). These results agree with the three major haplogroups observed in the network. The first group includes the nine localities that are in the province of Buenos Aires, Desaguadero River and the locality located in Uruguay, all leading to the Atlantic and forming part of the exorheic system basins. Surprisingly, this group is also composed of two endorheic adjacent rivers. The second group includes the two endorheic localities in the center of Argentina and other four nearby localities, some of which only recently became exorheic by anthropogenic actions (Cardoso et al., 2015). Finally, the third group is distributed more to the north of Argentina, covering the remaining six locations, two of them from exorheic basins.

We performed four AMOVA analyses to test different scenarios underlying population structure (Table 2). When we tested for the hydrographic system (Table 2‐a), for the basin type (Table 2‐b) and for the altitude (Table 2‐c), we found a low FCT (0.12, 0.06, −0.01, respectively).

**TABLE 2:** Analysis of molecular variance (AMOVA) based on the mitochondrial gene COI for *Jenynsia lineata*

Structure	Source of variation	*df*	ss	vc	%	fi
(a) By hydrographic system	Among groups	7	201.03	0.35	12.36	0.12
(L1 L2 L3 L5 L14 L15 L16) (L19) (L8 L14)	Among populations within groups	11	230.96	1.72	59.99	0.68
(L6 L9) (L13) (L7 L8) (L10 L11 L12) (L17)	Within populations	202	160.61	0.79	27.65	0.72
(b) by Basin	Among groups	1	41.74	0.16	5.66	0.06*
(L1 L2 L3 L5 L6 L9 L14 L15 L16 L19 L20)	Among populations within groups	17	390.25	1. 92	66.76	0.71
(L4 L7 L8 L10 L11 L12 L13 L17 L18)	Within populations	202	160.61	0. 79	27.58	0.72
(c) by Altitude	Among groups	4	99.55	−0.03	−1.25	−0.01*
(L1 L2 L3 L6 L14 L16 L19 L20) (L5 L15 L18)	Among populations within groups	14	357.44	2.03	72.81	0.72
(L9 L17) (L4 L7 L8 L12 L13) (L10 L11)	Within populations	202	160.61	0.79	28.45	0.71

Note

We tested for the structuration effect of (a) hydrographic system, (b) basin type, and (c) altitude.

*df* = degrees of freedom; ss = sum of squares; vc = variance components; % = percentage of variation; and fi = fixation indices. Locality codes are as in the Table 1.

* *p* non significant.

### 3.2 Testing explanatory factors with db‐RDA

To assess the association between the genetic structure and the variables that underlie the patterns of IBD, IBB, and IBE, we performed multiple regression analyses using the db‐RDA method. Our response variable was the genetic structure expressed by the locality pairwise‐FST matrix. The explanatory variables were as follows: (a) the geographical distance between localities as indicated by the first six axes of a PCNM on the geographical distance matrix where each axis was considered as separate variables (representing IBD); (b) the basin and (c) system type (both representing IBB), as categorical variables; (d) the altitude expressed as meters above sea level; and (e) latitude expressed by decimal coordinates (both representing IBE).

To select the set of variables that best explains the population structuration of *J. lineata*, we proceeded as follows: First, the full model was considered with all the variables, but it was not significant (*p* =.182). We also ran a db‐RDA on nested models to identify the best model (AIC = −132.68). However, the best model only considered one variable (third axis of PCNM) and it was not significant (*p* =.091).

**FIGURE 4:**
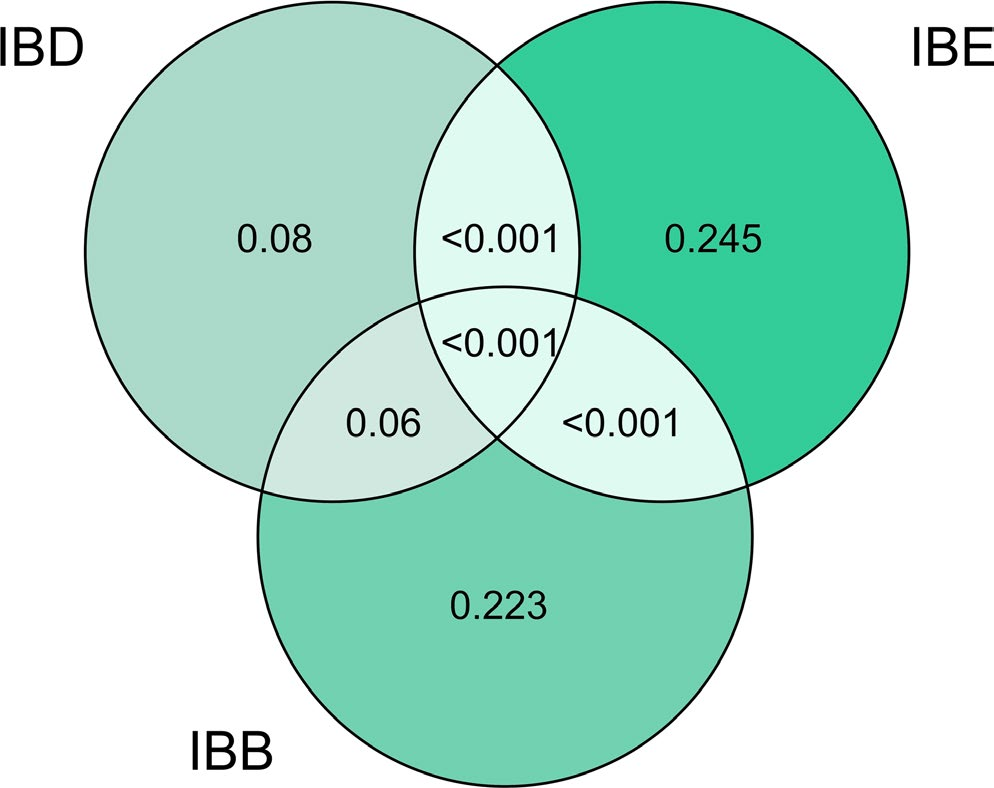
Variance partitioning analysis of the db‐RDA results. The variation of the locality pairwise genetic differentiation (FST) is explained by the variables underlying IBD (geographical distance), IBB (basin type) and IBE (altitude), and their interactions. The variance explained is indicated by AIC

As a consequence, we tested all possible combinations among variables deleting one by one from the full model. Then, we filtered only the models with a significant *p* (indicated in yellow, Table S5). Out of all these combinations with significant *p*, the optimal model for explaining the genetic structure was chosen according to AIC (−136.0251; Table S5). This model was able to explain up to 65% of the genetic structure (*p* =.01, *R* ^2^ = 0.6486) and contained three variables out of the initial 9: the geographical distance expressed as the third axes of the PCNM analysis (variable 1), basin type (variable 2) and altitude (variable 3). The variance partitioning analysis was performed only on the variables that were included in the optimal model, and we grouped them according to the pattern of isolation they generate: IBD, IBB, or IBE. The results showed that the variable driving IBE (altitude) explained 25% of the variance of the genetic structure, whereas the variable IBB (basin type) explained 22%. On the other hand, corroborating the Mantel test results, geographical distance (IBD) alone did not explain (*p* nonsignificant) the genetic variation (Figure 4). The interaction of the three explanatory variables explained only 3% of the response variable.

## 4 DISCUSSION

In the present work, we inferred the population genetic structure of *J. lineata* from across 19 localities distributed throughout Argentina, part of Uruguay and Southern Brazil. We tested whether multiple variables are driving the genetic structuration in this species. Among the most common classic patterns of isolation in landscape genetics, we have detected genetic footprints of IBB and IBE.

### 4.1 Isolation by distance

A higher genetic differentiation is expected for populations that are geographically far away from each other because their gene flow is reduced as compared to populations that are geographically close (IBD) (Wright, 1943). The degree of connectivity among populations is also related to the dispersal capacity of a given species, which is often related to body size (small fish have a higher migratory energy cost per unit distance than larger fish (Bernatchez & Dodson, 1987)). As explained by Peterson and Denno (1998), species with high dispersion usually do not present IBD because they have too high of a gene flow that consequently homogenizes populations independently of how distant they are. On the other hand, populations of poor dispersers are commonly so strongly structured that any potential role of geographical distance is overcome. Regarding our results, since it is a small fish, *J. lineata* should have a limited dispersal capacity, so its structuring could be due to other factors, such as population size, which affects the geographical distance, which we evidence in our analysis (Mantel test and db‐RDA) where we did not detect any IBD pattern in this species.

### 4.2 Isolation by environment

In tropical regions, where there is a great environmental heterogeneity, it is expected that habitat diversity plays an important role in the genetic structuration of species leading to population isolation by IBE. In this type of isolation, genetic differentiation increases with environmental differentiation, regardless of geographical distance (Wang & Bradburd, 2014). These environmental variables can be continuous, such as altitude, temperature, or humidity (Bradburd et al., 2013; Byars et al., 2009; Murphy et al., 2010), or discreet, as the type of substrate or vegetation cover (Andrew et al., 2012; Jardim de Queiroz et al., 2017). One of the reasons to choose *J. lineata* as a case study was its great tolerance to environmental factors. This species is found in freshwaters and marine environments covering a wide distribution range (Amorim, 2018; Calviño & Alonso, 2016). On the other hand, the wide distribution of the species as well as the complex network of haplotypes could also be the result of anthropic factors, such as human introductions (Arratia et al., 1983; Ghedotti & Weitzman, 1996) or environmental pollutants (Bickham et al., 2000). This has led some authors to suggest that *J. lineata* is actually a species complex that contains cryptic species (Aguilera et al., 2013; Ghedotti & Weitzman, 1996).

Regarding the results of this work, applying a db‐RDA, we tested latitude and altitude as environmental explanatory variables (IBE) to see whether they play an important role in the structuration of populations of this species. As explained before, features as altitudinal or latitudinal clines are important landscape characteristics that affect the proportion of suitable habitats, influence migration patterns and ultimately genetic divergence of populations (Giordano et al., 2007; Manel et al., 2003).

The landscape, including physical conditions and the biotic environment, can profoundly change due to altitude, creating gradients of temperature, humidity, biological community composition, etc (Kessler et al., 2001; Linden et al., 2014; Meier et al., 2010). For *Jenynsia lineata*, altitude was significant in conjunction with the type of basin and remained in the final reduced best model. Many hypotheses have been proposed to explain the changes in diversity associated with altitude, based on some ecological factors, such as reduction of available area and environmental complexity, great severity of climatic conditions, reduction in the diversity of available resources and decrease in productivity (Huston, 1994). Even so, there is no consensus on how these factors vary and interact with environmental variables in generating the observed diversity patterns (Nogués‐Bravo et al., 2008).

The other environmental variable that we studied as an example of the IBE was latitude. It has been seen recently that marine fish speciation is faster in geographic regions with lower species' richness (Rabosky et al., 2018), and for *Jenynsia lineata*, latitude did not play a significant role in its population structure, which could be because we did not include a large latitude range in our study. Despite the checked LGD, our results did support a correlation of latitude and population structure.

### 4.3 Isolation by barriers

Biogeographic barriers, such as oceans, mountain ranges, waterfalls, and fragmentation of basins, prevent the exchange of species between regions. Historically, these barriers have been important factors in determining the composition of fauna and in promoting endemism (Rahel, 2007). This type of endorheic–exorheic basin transition phenomena has already been seen and studied in other parts of the world such as in the Malawi and Tanganyika Lakes in Africa (Berry et al., 2019), in the Douro (Cunha et al., 2019), Tagus (Karampaglidis et al., 2020), and Ebro Rivers in Spain (Soria‐Jáuregui et al., 2019), among others. Regarding fish, the Pleistocene climatic oscillations have been studied in Cyprinidae of the Qinghai–Tibet Plateau, where a high diversity and genetic structure was evidenced in the populations that inhabited endorheic basins (Liang et al., 2017). On the other hand, the Quinto River crosses a wide plain in central Argentina and continues to the Amarga Wetland. In this area, during the dry periods, the river used to disappear on the surface, behaving like an endorheic basin. However, naturally during high rainfall periods, the Quinto River drained a considerable area, reaching the provinces of Santa Fe, La Pampa, and Buenos Aires. As an exorheic river, the Quinto River occasionally came into contact with watercourses associated with the Salado basin in the province of Buenos Aires (Ceci & Coronado, 1981; Menni, 2004).

In this study, fragmentation in endorheic and exorheic rivers was taken into account allowing us to explain a large part of the genetic variation. Our analysis showed that *J. lineata* comprises three haplogroups—one of them was made up of localities with exorheic basins, and the other two were made up of localities with endorheic basins. We suggest then that the basin fragmentation is an important factor to explain the population structuring of this species.

The divergence of the population inhabiting endorheic and exorheic rivers could have been triggered by a potential partial isolation of the La Plata Basin and the near river basin and has been shown to act as a barrier to gene flow at population levels in *Jenynsia lineata*. The hypothesis we propose here relies on the possibility that in South America, the Plio‐Pleistocene epoch was characterized by cycles of dry to humid climatic changes in large amplitude (Rull, 2008). Our working hypothesis states that during the driest periods, the water flow of rivers may have been reduced up to the point of disconnecting some tributaries from the rest of the basin. This fragmentation–reconnection dynamic depended on the climatic fluctuations of the Plio‐Pleistocene (Ritter et al., 2017).

Two of the three haplogroups found in *J. lineata* include most of the endorheic localities in central and northern Argentina and some nearby exorheic localities. This and the fact that geographic distances did not show a significant role for this species support that the pattern of isolation between the haplogroups is partially due to a physical barrier. As expected, within these two haplogroups (colored in violet and green in Figure 3), we found that the exoreic localities that are included are very near to the endorheic localities. These are the results of the reconnections that were achieved between the endorheic and exoreic basins during the humid periods.

## 5 CONCLUSION AND LINKED CONSIDERATIONS

Most population studies focus on how a single mechanism could affect structuration or speciation in a group of organisms. Our results show that the process of fish population diversification in the La Plata Basin is complex and not limited to a single process. Using a general method, we demonstrated that interactions among several processes have had an impact on the population structuring of *J. lineata*, a freshwater fish in South America. These mechanisms include geographical distance that leads to IBD, physical barriers that lead to IBB, and likely adaptation to environmental conditions related to differences in water characteristics that lead to IBE (temperature, dissolved oxygen, substrate). Moreover, the analyses of variance partitioning allowed us to unravel the relative role of these variables and the importance of their interactions. Hence, we showed that IBB and IBE processes have explained almost 50% of the population structuration in *J. lineata*. The power of this methodologies was corroborated here that it can easily be implemented for any other species.

The impact of the basin fragmentation phenomenon (as IBB pattern) on the diversity of freshwater fishes is expected to be significant and also general for all freshwater organisms. The fact that the basin fragmentation–reconnection hypothesis states a periodic repetition of the events, it may well represent a major process by which population structuring or new species are periodically created and may spread all over the basin during river reconnection periods. Nonetheless, further research is needed to disentangle this challenging issue. We expect to find in future studies that this hypothesis will be corroborated in other fish species and freshwater organisms.

The impact found of the phenomenon of fragmentation of the basin highlights the importance of the recent anthropic modifications that alter the natural runoff of the rivers. Dams and the constructions of artificial aquatic channels cause obvious disturbances in rivers and are likely to modify the gene flow previously attributed to natural features. For example, the Quinto River, which was originally endorheic, was affected in recent years as it has reactivated a superficial connection between this river and the Salado River through the artificial channel Arturo Jauretche (Ministry of Infrastructure of the Province of Buenos Aires 2015). Due to this, it can no longer be considered as strictly endorheic.

During the last decade in South America, it was necessary to focus studies at the species level or higher taxonomic level (Briñoccoli et al., 2020; Cardoso et al., 2018; Jardim de Queiroz et al., 2020). This type of work continues to be important for major biodiversity conservation efforts. However, the opportunities for inherent short‐term evolutionary processes acting at the intraspecific level have not yet been fully studied. We argue that the multiple processes of structuring and diversity within a population must be evaluated to maintain the variety of evolutionary pathways and to allow the emergence of a new diversity. After all, if processes that generate population structuring are maintained over time, such processes can, in turn, generate new species.

## CONFLICT OF INTEREST

None declared.

## AUTHOR CONTRIBUTIONS

**Yanina Briñoccoli:** Data curation (equal); formal analysis (equal); resources (equal); software (equal); writing–original draft (equal). **Luiz Jardim de Queiroz:** Data curation (equal); formal analysis (equal); methodology (equal); software (equal); writing–original draft (equal). **Sergio Bogan:** Data curation (equal); formal analysis (equal); resources (equal); writing–review and editing (equal). **Ariel Paracampo:** Data curation (equal); formal analysis (equal); writing–review and editing (equal). **Paula Posadas:** Project administration (equal); writing–review and editing (equal). **Gustavo M. Somoza:** Project administration (equal); writing–review and editing (equal). **Juan Montoya‐Burgos:** Conceptualization (equal); data curation (equal); formal analysis (equal); funding acquisition (equal); investigation (equal); project administration (equal); resources (equal); supervision (equal); writing–original draft (equal); writing–review and editing (equal). **Yamila P. Cardoso:** Conceptualization (equal); data curation (equal); formal analysis (equal); funding acquisition (equal); investigation (equal); methodology (equal); project administration (equal); resources (equal); supervision (equal); visualization (equal); writing–original draft (equal); writing–review and editing (equal).

## Supporting information

Supplementary Material

## Data Availability

The data used for this manuscript are openly available on GenBank under accession numbers: MN840645 ‐ MN840824.
