# Supplementary material for: Processes that drive the population structuring of Jenynsia lineata (Cyprinidontiformes, Anablepidae) in the La Plata Basin
Source: Ecol Evol. 2021 May 8;11(11):6119–32. doi: 10.1002/ece3.7427 (PMC8207347; doi:10.1002/ece3.7427)
Supplement: Supplementary file 1 — Supplementary Material [file ECE3-11-6119-s001.pdf]

**Processes that drive the population structuring of *Jenynsia lineata* (Cyprinidontiformes, Anablepidae) in the La Plata Basin**

Briñoccoli, YF; Jardim de Queiroz, L; Bogan, S; Paracampo, A; Posadas, PE, Somoza, GM; Montoya-Burgos, JI; Cardoso, YP

**Supporting Information**

**Figure S1.** Haplotype networks, colored by: A) Basin type, B) System and C) Altitude.

**Table S1.** Sampling localities, sub-localities and genetic diversity indices based on the COI gene for *Jenynsia lineata* .

**Table S2.** Summary of the sequences and collection locations of the specimens included in this study.

**Table S3.** Estimates of Evolutionary Divergence over Sequence Pairs between Groups in *Jenynsia lineata* .

**Table S4.** SAMOVA analyses based on the COI gene of *Jenynsia lineata* . a) with geographic distance from river courses; b) with geographic distance in straight-line

**Table S5.** Combinations of models tested for db-RDA analysis.

**Figure S1.** Haplotype networks, colored by: A) Basin type, B) System and C) Altitude.

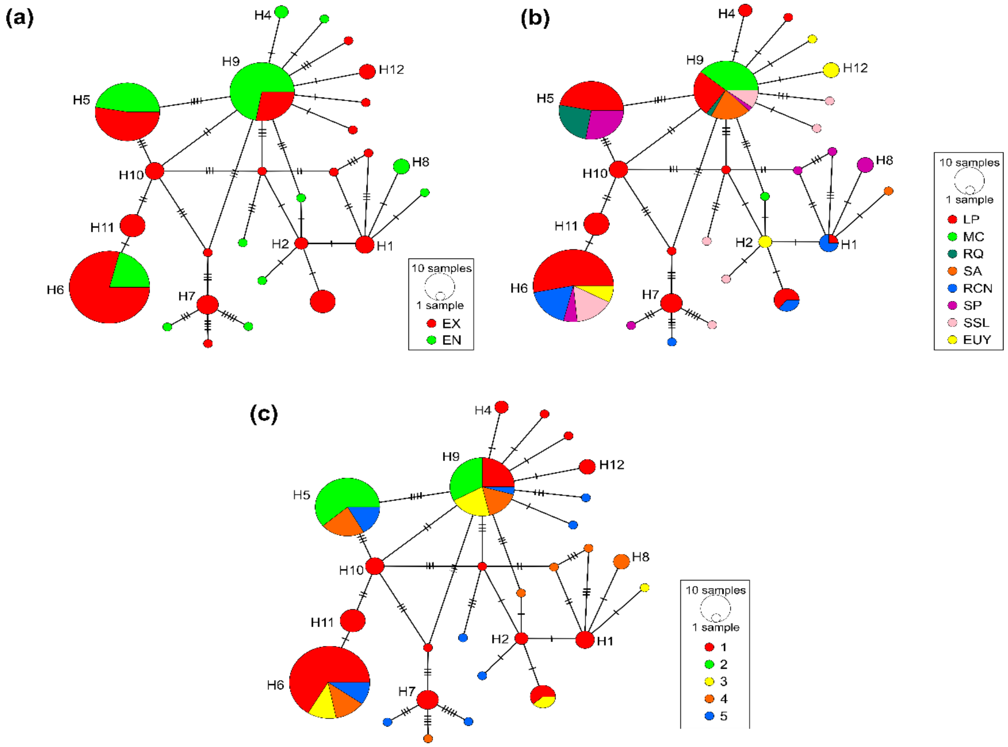

**Processes that drive the population structuring of *Jenynsia lineata* (Cyprinidontiformes, Anablepidae) in the La Plata Basin**

Briñoccoli, YF; Jardim de Queiroz, L; Bogan, S; Paracampo, A; Posadas, PE, Somoza, GM; Montoya-Burgos, JI; Cardoso, YP

**Supporting Information**

**Figure S1.** Haplotype networks, colored by: A) Basin type, B) System and C) Altitude.

**Table S1.** Sampling localities, sub-localities and genetic diversity indices based on the COI gene for *Jenynsia lineata*.

**Table S2.** Summary of the sequences and collection locations of the specimens included in this study.

**Table S3.** Estimates of Evolutionary Divergence over Sequence Pairs between Groups in *Jenynsia lineata*.
**Table S4.** SAMOVA analyses based on the COI gene of *Jenynsia lineata*. a) with geographic distance from river courses; b) with geographic distance in straight-line

**Table S5.** Combinations of models tested for db-RDA analysis.

**Table S1.** Sampling localities, sub-localities and genetic diversity indices based on the COI gene for *J. lineata*.

| System/ Basin | Location | Sublocation | Long | Lat | n | Altitude group | h | s | pi | Tajima's D | Gene diversity |
|---|---|---|---|---|---|---|---|---|---|---|---|
| **La Plata** | **1** | | | | **22** | 1 | **5** | **9** | **1.472** | **-1.353*** | **1.0000** |
| **EX** | | 1A | -59.248467 | -34.677583 | 2 | | | | | | |
| | | 1B | -58.477467 | -34.384967 | 2 | | | | | | |
| | | 1C | -58.485383 | -34.385417 | 2 | | | | | | |
| | | 1D | -58.998469 | -34.187308 | 2 | | | | | | |
| | | 1E | -59.5281 | -34.190578 | 2 | | | | | | |
| | | 1F | -57.774733 | -34.961578 | 1 | | | | | | |
| | | 1G | -57.625403 | -35.42652 | 3 | | | | | | |
| | | **1H** | **-58.11719** | **-34.782692** | 8 | | | | | | |
| | **2** | | | | **16** | 1 | **4** | **6** | **1.833** | **0.047*** | **1.0000** |
| | | **2A** | **-57.997858** | **-35.617486** | 4 | | | | | | |
| | | 2B | -58.0894 | -35.5411 | 1 | | | | | | |
| | | 2C | -57.879194 | -35.779194 | 4 | | | | | | |
| | | 2D | -59.98 | -36.268 | 1 | | | | | | |
| | | 2E | -61.12982 | -33.87179 | 2 | | | | | | |
| | | 2F | -60.957167 | -34.611806 | 4 | | | | | | |
| | **3** | | | | **12** | 1 | **2** | **5** | **1.621** | **-0.077*** | **1.0000** |
| | | 3A | -61.601389 | -38.335 | 1 | | | | | | |
| | | 3B | -61.876694 | -38.536389 | 1 | | | | | | |

| | | | | | | | | | | | |
|---|---|---|---|---|---|---|---|---|---|---|---|
| | | **3C** | **-57.317256** | **-37.566633** | 10 | | | | | | |
| | **5** | | | | **15** | **2** | **1** | **1** | **0.133** | **-1.159*** | **1.0000** |
| | | 5A | -64.428667 | -33.200889 | 1 | | | | | | |
| | | **5B** | **-64.359861** | **-33.94333** | 14 | | | | | | |
| | **14** | | | | **14** | **1** | **3** | **3** | **1.033** | **0.291*** | **1.0000** |
| | | 14A | -60.619833 | -31.29383 | 2 | | | | | | |
| | | **14B** | **-60.757444** | **-31.657111** | 12 | | | | | | |
| | **15** | | | | **7** | **2** | **1** | **0** | **0** | **0*** | **1.0000*** |
| | | **15A** | **-61.785694** | **-33.26** | 6 | | | | | | |
| | | 15B | -60.603713 | -32.93637 | 1 | | | | | | |
| | **16** | | **-62.603713** | **-32.93637** | **13** | **1** | **2** | **2** | **0.923** | **1.214*** | **1.0000** |
| **Mar Chiquita** | **4** | | | | **10** | **4** | **2** | **2** | **0.4** | **-1.401** | **1.0000*** |
| **EX** | | 4A | -64.502242 | -31.420522 | 1 | | | | | | |
| | | **4B** | **-64.448817** | **-31.729583** | 7 | | | | | | |
| | | 4C | -30.85 | -68.98 | 2 | | | | | | |
| | **18** | | **-63.462222** | **-29.3756** | **12** | **2** | **1** | **0** | **0** | **0** | **1.0000** |
| **Río Colorado-Negro** | **6** | | **-64.106117** | **-38.976575** | **9** | 1 | **2** | **2** | **1** | **1.235*** | **1.0000** |
| **EX** | **9** | | | | **13** | 3 | **3** | **9** | **2.462** | **-0.596*** | **1.0000** |
| | | 9A | -64.4712 | -40.099931 | 1 | | | | | | |
| | | 9B | -63.610758 | -40.71105 | 3 | | | | | | |
| | | 9C | -66.57483 | -33.761467 | 1 | | | | | | |
| | | 9D | -67.129744 | -33.410572 | 2 | | | | | | |
| | | **9E** | **-66.709994** | **-34.86956** | 5 | | | | | | |
| | | 9F | -66.26275 | -32.94383 | 1 | 3 | | | | | |
| **Salar de Pipanaco** | **7** | | **-66.21297** | **-30.772233** | **6** | **4** | **4** | **3** | **1.267** | **-0.185*** | **1.0000*** |
| **EN** | **8** | | | | **5** | 4 | **3** | **8** | **4.4** | **1.027*** | **1.0000*** |
| | | 8A | -66.21468 | -29.95797 | 1 | | | | | | |
| | | 8B | -66.551183 | -28.59367 | 1 | | | | | | |
| | | **8C** | **-66.54917** | **-28.6494** | 3 | | | | | | |

| | | | | | | | | | | | |
|---|---|---|---|---|---|---|---|---|---|---|---|
| **Sierras de San Luis** | **10** | | **-65.721722** | **-32.415639** | **9** | **5** | **8** | **17** | **5.389** | **-0.674*** | **1.0000** |
| **EN** | **11** | | | | **14** | **5** | **2** | **6** | **2.967** | **2.055*** | **1.0000** |
| | | **11A** | **-65.46175** | **-32.810472** | 9 | | | | | | |
| | | 11B | -65.212908 | -32.340297 | 1 | | | | | | |
| | | 11C | -65.646444 | -32.541417 | 4 | | | | | | |
| | **12** | | **-65.913361** | **-32.396361** | **6** | **4** | **2** | **8** | **4.267** | **1.284*** | **1.0000*** |
| **Río Quinto** | **13** | | | | **12** | **4** | **1** | **9** | **1.5** | **-2.016** | **1.0000** |
| **EN** | | 13A | -65.87128 | -33.33183 | 1 | | | | | | |
| | | 13B | -65.209222 | -33.856683 | 3 | | | | | | |
| | | **13C** | **-65.79215** | **-33.440733** | 8 | | | | | | |
| **Salar Ambargasta EN** | **17** | | **-65.170667** | **-28.62328** | **14** | **3** | **2** | **1** | **0.143** | **-1.155*** | **1.0000** |
| **Este Uruguay** | **19** | | | | **12** | **1** | **5** | **8** | **2.833** | **0.278*** | **1.0000** |
| **EX** | | **20A** | **-53.436389** | **-33.689444** | 4 | | | | | | |
| | | 20B | -53.585833 | -34.5278 | 1 | | | | | | |
| | | 20C | -54.238889 | -34.489167 | 1 | | | | | | |
| | | 20D | -52.51111 | -31.9722 | 3 | | | | | | |
| | | 20E | -48.873889 | -28.477222 | 2 | | | | | | |
| | | 20F | -33.252698 | -54.387093 | 1 | | | | | | |
| | **TOTAL** | **52** | | | **221** | **4** | **32** | **5.1** | **1.728** | ***** | |

Long= longitude; Lat= latitude; n= sample size; h= number of haplotypes; s= N of observed sites with substitutions; pi= mean N of pairwise differences.

The sub-localities and geographical coordinates in bold were those chosen to represent the locality.

* p non significant

## Processes that drive the population structuring of *Jenynsia lineata* (Cyprinidontiformes, Anablepidae) in the La Plata Basin

Briñoccoli, YF; Jardim de Queiroz, L; Bogan, S; Paracampo, A; Posadas, PE, Somoza, GM; Montoya-Burgos, JI; Cardoso, YP

**Supporting Information**

**Figure S1.** Haplotype networks, colored by: A) Basin type, B) System and C) Altitude.

**Table S1.** Sampling localities, sub-localities and genetic diversity indices based on the COI gene for *Jenynsia lineata* .

**Table S2.** Summary of the sequences and collection locations of the specimens included in this study.

**Table S3.** Estimates of Evolutionary Divergence over Sequence Pairs between Groups in *Jenynsia lineata* .

**Table S4.** SAMOVA analyses based on the COI gene of *Jenynsia lineata* . a) with geographic distance from river courses; b) with geographic distance in straight-line

**Table S5.** Combinations of models tested for db-RDA analysis.

**Table S2.** Summary of the sequences and collection locations of the specimens included in this study.

| Sample code | Genbank code | X | Y | Country | Province | Locality Name |
|---|---|---|---|---|---|---|
| FHN-216 | MN840645 | -64,502242 | -31,420522 | Argentina | Cordoba | Rio Primero |
| FHN-217 | MN840646 | -64,448817 | -31,729583 | Argentina | Cordoba | Rio Segundo |
| FHN-219 | MN840647 | -64,448817 | -31,729583 | Argentina | Cordoba | Rio Segundo |
| FHN-220 | MN840648 | -64,448817 | -31,729583 | Argentina | Cordoba | Rio Segundo |
| FHN-1628 | MN840649 | -64,448817 | -31,729583 | Argentina | Cordoba | Rio Segundo |
| FHN-1629 | MN840650 | -64,448817 | -31,729583 | Argentina | Cordoba | Rio Segundo |
| FHN-1432 | MN840651 | -64,448817 | -31,729583 | Argentina | Cordoba | Rio Segundo |
| FHN-1433 | MN840652 | -64,448817 | -31,729583 | Argentina | Cordoba | Rio Segundo |
| FHN-3072 | MN840653 | -64,359861 | -33,94333 | Argentina | Cordoba | Rio Cuarto |
| FHN-3073 | MN840654 | -64,359861 | -33,94333 | Argentina | Cordoba | Rio Cuarto |
| FHN-358 | MN840655 | -64,359861 | -33,94333 | Argentina | Cordoba | Rio Cuarto |
| YC09499 | MN840656 | -64,359861 | -33,94333 | Argentina | Cordoba | Rio Cuarto |
| YC09631 | MN840657 | -64,359861 | -33,94333 | Argentina | Cordoba | Rio Cuarto |
| YC09632 | MN840658 | -64,359861 | -33,94333 | Argentina | Cordoba | Rio Cuarto |
| YC09633 | MN840659 | -64,359861 | -33,94333 | Argentina | Cordoba | Rio Cuarto |
| YC13-184 | MN840660 | -64,359861 | -33,94333 | Argentina | Cordoba | Rio Cuarto |
| YC13-185 | MN840661 | -64,359861 | -33,94333 | Argentina | Cordoba | Rio Cuarto |
| YC13-186 | MN840662 | -64,359861 | -33,94333 | Argentina | Cordoba | Rio Cuarto |
| AR11-1402 | MN840663 | -64,359861 | -33,94333 | Argentina | Cordoba | Rio Cuarto |
| AR11-1403 | MN840664 | -64,359861 | -33,94333 | Argentina | Cordoba | Rio Cuarto |
| AR11-1404 | MN840665 | -64,359861 | -33,94333 | Argentina | Cordoba | Rio Cuarto |
| AR11-1405 | MN840666 | -64,359861 | -33,94333 | Argentina | Cordoba | Rio Cuarto |
| AR11-1190 | MN840667 | -64,428667 | -33,200889 | Argentina | Cordoba | Rio Santa Catalina |
| AR11-1235 | MN840668 | -64,479817 | -34,196933 | Argentina | Cordoba | Rio Quinto |
| AR11-1236 | MN840669 | -60,957167 | -34,611806 | Argentina | Buenos Aires | Rio Salado en Junin |
| AR11-1237 | MN840670 | -60,957167 | -34,611806 | Argentina | Buenos Aires | Rio Salado en Junin |
| AR11-1238 | MN840671 | -60,957167 | -34,611806 | Argentina | Buenos Aires | Rio Salado en Junin |
| AR11-1239 | MN840672 | -60,957167 | -34,611806 | Argentina | Buenos Aires | Rio Salado en Junin |
| AR11-1240 | MN840673 | -60,619833 | -31,29383 | Argentina | Santa Fe | Arroyo Aguilar |
| AR11-1241 | MN840674 | -60,619833 | -31,29383 | Argentina | Santa Fe | Arroyo Aguilar |
| AR11-1290 | MN840675 | -63,402699 | -28,973317 | Argentina | Santiago del Estero | Rio Saladillo cerca de Los Telares |
| AR11-1257 | MN840676 | -65,170667 | -28,62328 | Argentina | Santiago del Estero | Rio Albigasta en Frias |
| AR11-1258 | MN840677 | -65,170667 | -28,62328 | Argentina | Santiago del Estero | Rio Albigasta en Frias |
| AR11-1259 | MN840678 | -65,170667 | -28,62328 | Argentina | Santiago del Estero | Rio Albigasta en Frias |
| AR11-1260 | MN840679 | -65,170667 | -28,62328 | Argentina | Santiago del Estero | Rio Albigasta en Frias |
| AR11-1261 | MN840680 | -65,170667 | -28,62328 | Argentina | Santiago del Estero | Rio Albigasta en Frias |
| AR11-1262 | MN840681 | -65,170667 | -28,62328 | Argentina | Santiago del Estero | Rio Albigasta en Frias |

| | | | | | | |
|---|---|---|---|---|---|---|
| AR11-1263 | MN840682 | -65,170667 | -28,62328 | Argentina | Santiago del Estero | Rio Albigasta en Frias |
| AR11-1264 | MN840683 | -65,170667 | -28,62328 | Argentina | Santiago del Estero | Rio Albigasta en Frias |
| AR11-1265 | MN840684 | -65,170667 | -28,62328 | Argentina | Santiago del Estero | Rio Albigasta en Frias |
| AR11-1266 | MN840685 | -65,170667 | -28,62328 | Argentina | Santiago del Estero | Rio Albigasta en Frias |
| AR11-1267 | MN840686 | -65,170667 | -28,62328 | Argentina | Santiago del Estero | Rio Albigasta en Frias |
| AR11-1268 | MN840687 | -65,170667 | -28,62328 | Argentina | Santiago del Estero | Rio Albigasta en Frias |
| AR11-1269 | MN840688 | -65,170667 | -28,62328 | Argentina | Santiago del Estero | Rio Albigasta en Frias |
| AR11-1270 | MN840689 | -65,170667 | -28,62328 | Argentina | Santiago del Estero | Rio Albigasta en Frias |
| FHN-3000 | MN840690 | -60,757444 | -31,657111 | Argentina | Santa Fe | Rio Saladillo en Santo Tome |
| FHN-3001 | MN840691 | -60,757444 | -31,657111 | Argentina | Santa Fe | Rio Saladillo en Santo Tome |
| FHN-3004 | MN840692 | -60,757444 | -31,657111 | Argentina | Santa Fe | Rio Saladillo en Santo Tome |
| FHN-3005 | MN840693 | -60,757444 | -31,657111 | Argentina | Santa Fe | Rio Saladillo en Santo Tome |
| FHN-3006 | MN840694 | -60,757444 | -31,657111 | Argentina | Santa Fe | Rio Saladillo en Santo Tome |
| FHN-3007 | MN840695 | -60,757444 | -31,657111 | Argentina | Santa Fe | Rio Saladillo en Santo Tome |
| FHN-3003 | MN840696 | -60,757444 | -31,657111 | Argentina | Santa Fe | Rio Saladillo en Santo Tome |
| FHN-3008 | MN840697 | -60,757444 | -31,657111 | Argentina | Santa Fe | Rio Saladillo en Santo Tome |
| FHN-3002 | MN840698 | -60,757444 | -31,657111 | Argentina | Santa Fe | Rio Saladillo en Santo Tome |
| FHN-211 | MN840699 | -60,757444 | -31,657111 | Argentina | Santa Fe | Rio Saladillo en Santo Tome |
| FHN-212 | MN840700 | -60,757444 | -31,657111 | Argentina | Santa Fe | Rio Saladillo en Santo Tome |
| FHN-150 | MN840701 | -60,757444 | -31,657111 | Argentina | Santa Fe | Rio Saladillo en Santo Tome |
| FHN-155 | MN840702 | -62,603713 | -32,93637 | Argentina | Santiago del Estero | Rio Salado |
| FHN-156 | MN840703 | -62,603713 | -32,93637 | Argentina | Santiago del Estero | Rio Salado |
| FHN-3009 | MN840704 | -62,603713 | -32,93637 | Argentina | Santiago del Estero | Rio Salado |
| FHN-3015 | MN840705 | -62,603713 | -32,93637 | Argentina | Santiago del Estero | Rio Salado |
| FHN-3018 | MN840706 | -62,603713 | -32,93637 | Argentina | Santiago del Estero | Rio Salado |
| FHN-3017 | MN840707 | -62,603713 | -32,93637 | Argentina | Santiago del Estero | Rio Salado |
| YC14-022 | MN840708 | -62,603713 | -32,93637 | Argentina | Santiago del Estero | Rio Salado |
| YC14-023 | MN840709 | -62,603713 | -32,93637 | Argentina | Santiago del Estero | Rio Salado |
| FHN-201 | MN840710 | -62,603713 | -32,93637 | Argentina | Santiago del Estero | Rio Salado |
| FHN-202 | MN840711 | -62,603713 | -32,93637 | Argentina | Santiago del Estero | Rio Salado |
| FHN-203 | MN840712 | -62,603713 | -32,93637 | Argentina | Santiago del Estero | Rio Salado |
| FHN-204 | MN840713 | -62,603713 | -32,93637 | Argentina | Santiago del Estero | Rio Salado |
| FHN-210 | MN840714 | -62,603713 | -32,93637 | Argentina | Santiago del Estero | Rio Salado |
| YC14-080 | MN840715 | -63,402699 | -28,973317 | Argentina | Santiago del Estero | Rio Saladillo cerca de Los Telares |
| YC13-812 | MN840716 | -63,402699 | -28,973317 | Argentina | Santiago del Estero | Rio Saladillo cerca de Los Telares |
| YC13-814 | MN840717 | -63,402699 | -28,973317 | Argentina | Santiago del Estero | Rio Saladillo cerca de Los Telares |
| YC13-815 | MN840718 | -63,402699 | -28,973317 | Argentina | Santiago del Estero | Rio Saladillo cerca de Los Telares |
| YC13-816 | MN840719 | -63,402699 | -28,973317 | Argentina | Santiago del Estero | Rio Saladillo cerca de Los Telares |
| YC13-817 | MN840720 | -63,402699 | -28,973317 | Argentina | Santiago del Estero | Rio Saladillo cerca de Los Telares |
| YC13-648 | MN840721 | -63,402699 | -28,973317 | Argentina | Santiago del Estero | Rio Saladillo cerca de Los Telares |
| YC13-649 | MN840722 | -63,402699 | -28,973317 | Argentina | Santiago del Estero | Rio Saladillo cerca de Los Telares |
| YC13-650 | MN840723 | -63,402699 | -28,973317 | Argentina | Santiago del Estero | Rio Saladillo cerca de Los Telares |
| YC13-651 | MN840724 | -63,402699 | -28,973317 | Argentina | Santiago del Estero | Rio Saladillo cerca de Los Telares |
| YC13-652 | MN840725 | -63,402699 | -28,973317 | Argentina | Santiago del Estero | Rio Saladillo cerca de Los Telares |
| YC13-653 | MN840726 | -66,57483 | -33,761467 | Argentina | San Luis | Arroyo Bebedero y ruta 11 |
| YC13-654 | MN840727 | -65,212908 | -32,340297 | Argentina | San Luis | Rio Conlara en Santa Rosa |
| YC13-658 | MN840728 | -58,485383 | -34,385417 | Argentina | Buenos Aires | Canal del Este. Tigre |
| YC13-664 | MN840729 | -58,485383 | -34,385417 | Argentina | Buenos Aires | Canal del Este. Tigre |
| YC13-686 | MN840730 | -66,54917 | -28,6494 | Argentina | La Rioja | Rio Colorado en Villa Mazan |
| YC13-687 | MN840731 | -66,54917 | -28,6494 | Argentina | La Rioja | Rio Colorado en Villa Mazan |

| | | | | | | |
|---|---|---|---|---|---|---|
| YC13-688 | MN840732 | -66,54917 | -28,6494 | Argentina | La Rioja | Rio Colorado en Villa Mazan |
| YC13-695 | MN840733 | -58,477467 | -34,384967 | Argentina | Buenos Aires | Banado arroyo Desaguadero |
| YC14-026 | MN840734 | -58,477467 | -34,384967 | Argentina | Buenos Aires | Banado arroyo Desaguadero |
| YC14-027 | MN840735 | -65,79215 | -33,440733 | Argentina | San Luis | Rio Quinto |
| YC14-029 | MN840736 | -65,79215 | -33,440733 | Argentina | San Luis | Rio Quinto |
| YC14-034 | MN840737 | -65,79215 | -33,440733 | Argentina | San Luis | Rio Quinto |
| YC14-159 | MN840738 | -65,79215 | -33,440733 | Argentina | San Luis | Rio Quinto |
| YC13-943 | MN840739 | -65,79215 | -33,440733 | Argentina | San Luis | Rio Quinto |
| YC13-944 | MN840740 | -65,79215 | -33,440733 | Argentina | San Luis | Rio Quinto |
| YC13-945 | MN840741 | -65,79215 | -33,440733 | Argentina | San Luis | Rio Quinto |
| FHN-175 | MN840742 | -66,709994 | -34,86956 | Argentina | San Luis | Rio Desaguadero y Ruta 146 |
| FHN-177 | MN840743 | -66,709994 | -34,86956 | Argentina | San Luis | Rio Desaguadero y Ruta 146 |
| FHN-178 | MN840744 | -66,709994 | -34,86956 | Argentina | San Luis | Rio Desaguadero y Ruta 146 |
| FHN-179 | MN840745 | -66,709994 | -34,86956 | Argentina | San Luis | Rio Desaguadero y Ruta 146 |
| FHN-181 | MN840746 | -66,709994 | -34,86956 | Argentina | San Luis | Rio Desaguadero y Ruta 146 |
| FHN-183 | MN840747 | -66,21297 | -30,772233 | Argentina | La Rioja | Arroyo el Cisco y ruta 79 |
| AR11-1331 | MN840748 | -66,21297 | -30,772233 | Argentina | La Rioja | Arroyo el Cisco y ruta 79 |
| FHN-184 | MN840749 | -66,21297 | -30,772233 | Argentina | La Rioja | Arroyo el Cisco y ruta 79 |
| YC13-183 | MN840750 | -66,21297 | -30,772233 | Argentina | La Rioja | Arroyo el Cisco y ruta 79 |
| AR15-033 | MN840751 | -66,21297 | -30,772233 | Argentina | La Rioja | Arroyo el Cisco y ruta 79 |
| AR15-034 | MN840752 | -66,21297 | -30,772233 | Argentina | La Rioja | Arroyo el Cisco y ruta 79 |
| AR15-1462 | MN840753 | -64,106117 | -38,976575 | Argentina | La Pampa | Rio Colorado en La Adela |
| AR15-1464 | MN840754 | -64,106117 | -38,976575 | Argentina | La Pampa | Rio Colorado en La Adela |
| AR15-1466 | MN840755 | -64,106117 | -38,976575 | Argentina | La Pampa | Rio Colorado en La Adela |
| AR15-1463 | MN840756 | -64,106117 | -38,976575 | Argentina | La Pampa | Rio Colorado en La Adela |
| AR15-1467 | MN840757 | -64,106117 | -38,976575 | Argentina | La Pampa | Rio Colorado en La Adela |
| AR15-1473 | MN840758 | -64,106117 | -38,976575 | Argentina | La Pampa | Rio Colorado en La Adela |
| AR15-1470 | MN840759 | -64,106117 | -38,976575 | Argentina | La Pampa | Rio Colorado en La Adela |
| AR15-1471 | MN840760 | -64,106117 | -38,976575 | Argentina | La Pampa | Rio Colorado en La Adela |
| AR15-1468 | MN840761 | -64,106117 | -38,976575 | Argentina | La Pampa | Rio Colorado en La Adela |
| AR15-1469 | MN840762 | -64,4712 | -40,099931 | Argentina | Rio Negro | Canal de riego del Rio Negro |
| AR15-1472 | MN840763 | -63,610758 | -40,71105 | Argentina | Rio Negro | Canal de riego del Rio Negro |
| AR15-1476 | MN840764 | -63,610758 | -40,71105 | Argentina | Rio Negro | Canal de riego del Rio Negro |
| YC13-487 | MN840765 | -63,610758 | -40,71105 | Argentina | Rio Negro | Canal de riego del Rio Negro |
| YC13-488 | MN840766 | -58,998469 | -34,187308 | Argentina | Buenos Aires | arroyo La Cruz |
| YC13-489 | MN840767 | -58,99847 | -34,187309 | Argentina | Buenos Aires | arroyo La Cruz |
| YC13-490 | MN840768 | -59,5281 | -34,190578 | Argentina | Buenos Aires | Tosquera 1 Termoelec M Belgrano |
| YC13-491 | MN840769 | -59,5281 | -34,190578 | Argentina | Buenos Aires | Tosquera 1 Termoelec M Belgrano |
| AR15-779 | MN840770 | -66,551183 | -28,59367 | Argentina | La Rioja | Rio Colorado |
| AR15-783 | MN840771 | -66,21468 | -29,95797 | Argentina | La Rioja | Los Nacimientos |
| AR15-787 | MN840772 | -57,774733 | -34,961578 | Argentina | Buenos Aires | El Pescado |
| AR15-788 | MN840773 | -57,625403 | -35,42652 | Argentina | Buenos Aires | Juan Blanco |
| AR15-789 | MN840774 | -57,625403 | -35,42652 | Argentina | Buenos Aires | Juan Blanco |
| AR15-781 | MN840775 | -57,625403 | -35,42652 | Argentina | Buenos Aires | Juan Blanco |
| AR15-782 | MN840776 | -57,997858 | -35,617486 | Argentina | Buenos Aires | Chascomus |
| AR15-790 | MN840777 | -57,997858 | -35,617486 | Argentina | Buenos Aires | Chascomus |
| AR15-791 | MN840778 | -57,997858 | -35,617486 | Argentina | Buenos Aires | Chascomus |
| AR15-784 | MN840779 | -57,997858 | -35,617486 | Argentina | Buenos Aires | Chascomus |
| AR15-785 | MN840780 | -61,785694 | -33,26 | Argentina | Santa Fe | Rio Carcarana cerca de Cruz Alta |
| AR15-793 | MN840781 | -61,785694 | -33,26 | Argentina | Santa Fe | Rio Carcarana cerca de Cruz Alta |

| | | | | | | |
|---|---|---|---|---|---|---|
| AR15-780 | MN840782 | -61,785694 | -33,26 | Argentina | Santa Fe | Rio Carcarana cerca de Cruz Alta |
| AR15-1309 | MN840783 | -61,785694 | -33,26 | Argentina | Santa Fe | Rio Carcarana cerca de Cruz Alta |
| AR15-1308 | MN840784 | -61,785694 | -33,26 | Argentina | Santa Fe | Rio Carcarana cerca de Cruz Alta |
| AR15-1311 | MN840785 | -61,785694 | -33,26 | Argentina | Santa Fe | Rio Carcarana cerca de Cruz Alta |
| AR15-1315 | MN840786 | -65,46175 | -32,810472 | Argentina | San Luis | Embalse San Felipe |
| AR15-1316 | MN840787 | -65,46175 | -32,810472 | Argentina | San Luis | Embalse San Felipe |
| AR15-1321 | MN840788 | -65,46175 | -32,810472 | Argentina | San Luis | Embalse San Felipe |
| AR15-1310 | MN840789 | -65,46175 | -32,810472 | Argentina | San Luis | Embalse San Felipe |
| AR15-1312 | MN840790 | -65,46175 | -32,810472 | Argentina | San Luis | Embalse San Felipe |
| AR15-1313 | MN840791 | -65,46175 | -32,810472 | Argentina | San Luis | Embalse San Felipe |
| AR15-1314 | MN840792 | -65,46175 | -32,810472 | Argentina | San Luis | Embalse San Felipe |
| AR15-1317 | MN840793 | -65,46175 | -32,810472 | Argentina | San Luis | Embalse San Felipe |
| AR15-1318 | MN840794 | -65,46175 | -32,810472 | Argentina | San Luis | Embalse San Felipe |
| AR15-1319 | MN840795 | -65,646444 | -32,541417 | Argentina | San Luis | Villa Praga |
| AR15-1320 | MN840796 | -65,646444 | -32,541417 | Argentina | San Luis | Villa Praga |
| AR15-997 | MN840797 | -65,646444 | -32,541417 | Argentina | San Luis | Villa Praga |
| AR15-998 | MN840798 | -65,646444 | -32,541417 | Argentina | San Luis | Villa Praga |
| AR15-999 | MN840799 | -65,721722 | -32,415639 | Argentina | San Luis | Embalse La Huertita |
| AR15-1000 | MN840800 | -65,721722 | -32,415639 | Argentina | San Luis | Embalse La Huertita |
| AR15-988 | MN840801 | -65,721722 | -32,415639 | Argentina | San Luis | Embalse La Huertita |
| AR15-989 | MN840802 | -65,721722 | -32,415639 | Argentina | San Luis | Embalse La Huertita |
| AR15-990 | MN840803 | -65,721722 | -32,415639 | Argentina | San Luis | Embalse La Huertita |
| AR15-991 | MN840804 | -65,721722 | -32,415639 | Argentina | San Luis | Embalse La Huertita |
| AR15-992 | MN840805 | -65,721722 | -32,415639 | Argentina | San Luis | Embalse La Huertita |
| AR15-993 | MN840806 | -65,721722 | -32,415639 | Argentina | San Luis | Embalse La Huertita |
| AR15-994 | MN840807 | -65,721722 | -32,415639 | Argentina | San Luis | Embalse La Huertita |
| AR15-995 | MN840808 | -65,913361 | -32,396361 | Argentina | San Luis | Rio Lujan aguas abajo del dique |
| FHN-102 | MN840809 | -65,913361 | -32,396361 | Argentina | San Luis | Rio Lujan aguas abajo del dique |
| YC13-810 | MN840810 | -65,209222 | -33,856683 | Argentina | San Luis | Rio Quinto en Bajo Vara |
| YC13-811 | MN840811 | -65,209222 | -33,856683 | Argentina | San Luis | Rio Quinto en Bajo Vara |
| FHN-365 | MN840812 | -65,209222 | -33,856683 | Argentina | San Luis | Rio Quinto en Bajo Vara |
| YC13-573 | MN840813 | -67,129744 | -33,410572 | Argentina | San Luis | Rio Desaguadero |
| FHN-043 | MN840814 | -67,129744 | -33,410572 | Argentina | San Luis | Rio Desaguadero |
| YC-959 | MN840815 | -65,913361 | -32,396361 | Argentina | San Luis | Rio Lujan aguas abajo del dique |
| YC-960 | MN840816 | -65,913361 | -32,396361 | Argentina | San Luis | Rio Lujan aguas abajo del dique |
| YC13-827 | MN840817 | -65,913361 | -32,396361 | Argentina | San Luis | Rio Lujan aguas abajo del dique |
| YC13-873 | MN840818 | -65,913361 | -32,396361 | Argentina | San Luis | Rio Lujan aguas abajo del dique |
| YC13-874 | MN840819 | -66,26275 | -32,94383 | Argentina | San Luis | Rio Nogoli |
| FHN-3651 | MN840820 | -65,209222 | -33,856683 | Argentina | San Luis | Embalse Paso de las Carretas |
| YC13-828 | MN840821 | -59,248467 | -34,677583 | Argentina | Buenos Aires | Arroyo Balta sobre ruta 5 |
| FHN-3816 | MN840822 | -59,248467 | -34,677583 | Argentina | Buenos Aires | Arroyo Balta sobre ruta 5 |
| YC-950B | MN840823 | -61,12982 | -33,87179 | Argentina | Buenos Aires | Colon |
| YC-952B | MN840824 | -61,12982 | -33,87179 | Argentina | Buenos Aires | Colon |

**Processes that drive the population structuring of *Jenynsia lineata* (Cyprinidontiformes, Anablepidae) in the La Plata Basin**

Briñoccoli, YF; Jardim de Queiroz, L; Bogan, S; Paracampo, A; Posadas, PE, Somoza, GM; Montoya-Burgos, JI; Cardoso, YP

**Supporting Information**

**Figure S1.** Haplotype networks, colored by: A) Basin type, B) System and C) Altitude.

**Table S1.** Sampling localities, sub-localities and genetic diversity indices based on the COI gene for *Jenynsia lineata* .

**Table S2.** Summary of the sequences and collection locations of the specimens included in this study.

**Table S3.** Estimates of Evolutionary Divergence over Sequence Pairs between Groups in *Jenynsia lineata* .

**Table S4.** SAMOVA analyses based on the COI gene of *Jenynsia lineata* . a) with geographic distance from river courses; b) with geographic distance in straight-line

**Table S5.** Combinations of models tested for db-RDA analysis.

**Table S3.** Estimates of Evolutionary Divergence over Sequence Pairs between Groups in *Jenynsia lineata* . Analyses were conducted using the Kimura 2-parameter model.

| | 1 | 2 | 3 | 4 | 5 | 6 | 7 | 8 | 9 | 10 | 11 | 12 | 13 | 14 | 15 | 16 | 17 | 18 | 19 | 20 |
|---|---|---|---|---|---|---|---|---|---|---|---|---|---|---|---|---|---|---|---|---|
| 1 | 0 | | | | | | | | | | | | | | | | | | | |
| 2 | 0,002 | 0 | | | | | | | | | | | | | | | | | | |
| 3 | 0,002 | 0,003 | 0 | | | | | | | | | | | | | | | | | |
| 4 | 0,01 | 0,01 | 0,01 | 0 | | | | | | | | | | | | | | | | |
| 5 | 0,01 | 0,011 | 0,009 | 0,016 | 0 | | | | | | | | | | | | | | | |
| 6 | 0,001 | 0,002 | 0,002 | 0,01 | 0,011 | 0 | | | | | | | | | | | | | | |
| 7 | 0,011 | 0,012 | 0,012 | 0,002 | 0,017 | 0,012 | 0 | | | | | | | | | | | | | |
| 8 | 0,007 | 0,008 | 0,006 | 0,014 | 0,005 | 0,007 | 0,015 | 0 | | | | | | | | | | | | |
| 9 | 0,003 | 0,003 | 0,003 | 0,011 | 0,01 | 0,003 | 0,012 | 0,008 | 0 | | | | | | | | | | | |
| 10 | 0,006 | 0,007 | 0,006 | 0,012 | 0,009 | 0,006 | 0,013 | 0,009 | 0,007 | 0 | | | | | | | | | | |
| 11 | 0,007 | 0,007 | 0,006 | 0,014 | 0,004 | 0,007 | 0,015 | 0,005 | 0,007 | 0,008 | 0 | | | | | | | | | |
| 12 | 0,004 | 0,005 | 0,004 | 0,006 | 0,012 | 0,004 | 0,008 | 0,009 | 0,005 | 0,008 | 0,009 | 0 | | | | | | | | |
| 13 | 0,01 | 0,011 | 0,009 | 0,015 | 0,001 | 0,011 | 0,016 | 0,006 | 0,01 | 0,009 | 0,004 | 0,011 | 0 | | | | | | | |
| 14 | 0,01 | 0,01 | 0,01 | 0,002 | 0,015 | 0,01 | 0,003 | 0,013 | 0,01 | 0,011 | 0,013 | 0,007 | 0,014 | 0 | | | | | | |
| 15 | 0,01 | 0,011 | 0,009 | 0,016 | 0 | 0,011 | 0,017 | 0,005 | 0,01 | 0,009 | 0,004 | 0,012 | 0,001 | 0,015 | 0 | | | | | |
| 16 | 0,01 | 0,011 | 0,011 | 0,001 | 0,016 | 0,011 | 0,002 | 0,014 | 0,011 | 0,012 | 0,014 | 0,007 | 0,015 | 0,002 | 0,016 | 0 | | | | |
| 17 | 0,011 | 0,011 | 0,011 | 0,001 | 0,016 | 0,011 | 0,003 | 0,014 | 0,011 | 0,012 | 0,014 | 0,007 | 0,015 | 0,002 | 0,016 | 0,001 | 0 | | | |
| 18 | 0,01 | 0,011 | 0,011 | 0 | 0,016 | 0,011 | 0,002 | 0,014 | 0,011 | 0,012 | 0,014 | 0,007 | 0,015 | 0,002 | 0,016 | 0,001 | 0 | 0 | | |
| 19 | 0,005 | 0,006 | 0,006 | 0,011 | 0,011 | 0,006 | 0,012 | 0,009 | 0,006 | 0,009 | 0,009 | 0,007 | 0,011 | 0,01 | 0,011 | 0,011 | 0,011 | 0,011 | 0 | |
| 20 | 0,004 | 0,004 | 0,005 | 0,011 | 0,012 | 0,004 | 0,013 | 0,009 | 0,005 | 0,008 | 0,009 | 0,006 | 0,012 | 0,011 | 0,012 | 0,012 | 0,012 | 0,012 | 0,006 | 0 |

**Processes that drive the population structuring of *Jenynsia lineata* (Cyprinidontiformes, Anablepidae) in the La Plata Basin**

Briñoccoli, YF; Jardim de Queiroz, L; Bogan, S; Paracampo, A; Posadas, PE, Somoza, GM; Montoya-Burgos, JI; Cardoso, YP

**Supporting Information**

**Figure S1.** Haplotype networks, colored by: A) Basin type, B) System and C) Altitude.

**Table S1.** Sampling localities, sub-localities and genetic diversity indices based on the COI gene for *Jenynsia lineata*.

**Table S2.** Summary of the sequences and collection locations of the specimens included in this study.

**Table S3.** Estimates of Evolutionary Divergence over Sequence Pairs between Groups in *Jenynsia lineata*.
**Table S4.** SAMOVA analyses based on the COI gene of *Jenynsia lineata*. a) with geographic distance from river courses; b) with geographic distance in straight-line

**Table S5.** Combinations of models tested for db-RDA analysis.

**Table S4.** SAMOVA analyses based on the COI gene of *Jenynsia lineata. a)* with geographic distance from river courses; b) with geographic distance in straight-line

a)

| K | FCT | Structure |
|---|---|---|
| 2 | -0.143 | (L1 L2 L3 L4 L5 L7 L8 L9 L10 L11 L12 L13 L15 L14 L16 L17 L18 L19) (L6) |
| 3 | 0.744 | (L1 L2 L3 L6 L9 L10 L12 L19) (L5 L8 L11 L13 L15) (L4 L7 L14 L16 L17 L18) |
| 4 | 0.743 | (L1 L2 L3 L6 L9 L10 L12 L19) (L5 L8 L11 L13 L15) (L4 L14 L16 L17 L18) (L7) |
| 5 | 0.744 | (L1 L2 L3 L6 L9 L10 L12 L19) (L5 L13 L15) (L4 L7 L14 L16 L17 L18) (L8) (L11) |
| 6 | 0.744 | (L1 L2 L3 L6 L9 L19) (L5 L13 L15) (L4 L7 L14 L16 L17 L18) (L8 L11) (L10) (L12) |
| 7 | 0.727 | (L1 L2 L3 L6 L9 L10 L12 L19) (L5 L8 L13 L15) (L17 L18) (L7) (L11) (L14 L16) (L4) |
| 8 | 0.744 | (L1 L2 L3 L6 L9 L19) (L5 L13 L15) (L4 L14 L16 L17 L18) (L7) (L8) (L10) (L11) (L12) |
| 9 | 0.749 | (L1 L2 L3 L6 L9) (L5 L13 L15) (L4 L14 L16 L17 L18) (L7) (L8) (L11) (L10) (L12) (L19) |
| 10 | 0.730 | (L1 L2 L3 L6 L19) (L5 L13 L15) (L4 L17 L18) (L7) (L8) (L11) (L10) (L12) (L9) (L14 L16) |

b)

| K | FCT | Structure |
|---|---|---|
| 2 | 0.662 | (L1 L2 L3 L5 L6 L8 L9 L10 L11 L12 L13 L15 L19) (L4 L7 L14 L16 L17 L18) |
| 3 | 0.744 | (L1 L2 L3 L6 L9 L10 L12 L19) (L5 L8 L11 L13 L15) (L4 L7 L14 L16 L17 L18) |
| 4 | 0.742 | (L1 L2 L3 L6 L9 L10 L12 L19) (L5 L11 L13 L15) (L4 L7 L14 L16 L17 L18) (L8) |
| 5 | 0.740 | (L1 L2 L3 L6 L8 L9 L10 L12 L19) (L5 L13 L15) (L4 L14 L16 L17 L18) (L7) (L11) |
| 6 | 0.746 | (L1 L2 L3 L6 L9 L12 L19) (L5 L13 L15) (L4 L14 L16 L17 L18) (L8 L11) (L7) (L10) |
| 7 | 0.743 | (L1 L2 L3 L6 L9 L12 L19) (L5 L13 L15) (L4 L14 L16 L17 L18) (L8) (L7) (L10) (L11) |
| 8 | 0.744 | (L1 L2 L3 L6 L9 L19) (L5 L13 L15) (L4 L14 L16 L17 L19) (L8) (L7) (L10) (L11) (L12) |
| 9 | 0.744 | (L1 L2 L3 L6 L9 L12) (L5 L13 L15) (L4 L17 L18) (L8) (L7) (L10) (L11 L14 L16) (L19) |
| 10 | 0.749 | (L1 L2 L3 L6 L9) (L5 L13 L15) (L4 L17 L18) (L8) (L7) (L10) (L11) (L12) (L19) (L14 L16) |

L "number" means: location and number (a total of 19 locations)

**Processes that drive the population structuring of *Jenynsia lineata* (Cyprinidontiformes, Anablepidae) in the La Plata Basin**

Briñoccoli, YF; Jardim de Queiroz, L; Bogan, S; Paracampo, A; Posadas, PE, Somoza, GM; Montoya-Burgos, JI; Cardoso, YP

**Supporting Information**

**Figure S1.** Haplotype networks, colored by: A) Basin type, B) System and C) Altitude.

**Table S1.** Sampling localities, sub-localities and genetic diversity indices based on the COI gene for *Jenynsia lineata* .

**Table S2.** Summary of the sequences and collection locations of the specimens included in this study.

**Table S3.** Estimates of Evolutionary Divergence over Sequence Pairs between Groups in *Jenynsia lineata* .

**Table S4.** SAMOVA analyses based on the COI gene of *Jenynsia lineata* . a) with geographic distance from river courses; b) with geographic distance in straight-line

**Table S5.** Combinations of models tested for db-RDA analysis.

**Table S5.** Combinations of models tested for db-RDA analysis.

| | AIC (green the highest values) | R | p value (yellow signiticative) | significatives VE | Model analyzed |
|---|---|---|---|---|---|
| **Full model** | -132.6248 | 0.686 | 0.182 | 0 | gen_dist ~ scale(geo1) + scale(geo2)+ scale(geo3)+ scale(geo4)+ scale(geo5)+ scale(geo6) + scale(Altitude) + scale(latitude) + BasinType + system |
| **Best model** | -132.68 | | 0.091 | 0 | gen_dist ~ scale(geo3) |
| *More tests with different combinations of explanatory variables* | | | | | |
| **Without basin** | -125.6033 | 1.029 | 0.645 | 0 | gen_dist, ~ scale(geo1)+ scale(geo2) + scale(geo3) + scale(geo4) + scale(geo5)+ scale(geo6), scale(Altitude)+ scale(latitude), ~system |
| **Without geos*** | -133.257 | 0.749 | 0.048 | altitud and basin | gen_dist,~ scale(Altitude)+ scale(latitude), ~BasinType + system |
| **Without system** | -133.8333 | 0.642 | 0.085 | altitud and basin | gen_dist ~ scale(geo1) + scale(geo2) + scale(geo3) + scale(geo4) + scale(geo5) + scale(geo6) + scale(Altitude) + scale(latitude) + BasinType |
| from here we extract one per one variables from the model without system (we remove those with the highest p-value) | | | | | |
| without geo1 | -134.579 | 0.62 | 0.051 | geo3, basin and altitude | gen_dist, ~ scale(geo2) + scale(geo3) + scale(geo4) + scale(geo5)+ scale(geo6), ~ scale(latitude)+scale(Altitude), ~BasinType |
| without geo5 | -134.9312 | 0.613 | 0.038 | altitud and basin | gen_dist, ~ scale(geo2) + scale(geo3) + scale(geo4) + scale(geo6), ~ scale(latitude)+scale(Altitude), ~BasinType |
| without latitude | -133.8912 | 0.673 | 0.046 | altitud and basin | gen_dist, ~ scale(geo2) + scale(geo3) + scale(geo4) + scale(geo6), ~ scale(Altitude), ~BasinType |
| without geo6 | -134.9192 | 0.6462 | 0.024 | altitud and basin | gen_dist, ~ scale(geo2) + scale(geo3) + scale(geo4), ~ scale(Altitude), ~BasinType |
| without geo2 | -135.8216 | 0.626 | 0.015 | altitud and basin | gen_dist, ~ scale(geo3) + scale(geo4), ~ scale(Altitude), ~BasinType |
| without geo4 | **-136.0251** | 0.6486 | 0.01 | altitud and basin | gen_dist, ~ scale(geo3), ~ scale(Altitude), ~BasinType |
| without geo3 | -135.2652 | 0.729 | 0.013 | altitud and basin | gen_dist, ~ scale(Altitude) + BasinType |
| **without altitude** | -131.867 | 0.705 | 0.178 | geo3 and basin | gen_dist, ~scale(geo1)+ scale(geo2)+ scale(geo3)+ scale(geo4)+ scale(geo5)+ scale(geo6), ~ scale(latitude), ~BasinType + system |
| extracted variables one per one from the model without altitude (we remove those with the highest p-value) | | | | | |
| without system | -128.2051 | 0.8882 | 0.39 | 0 | en_dist, ~ scale(geo1)+ scale(geo2)+ scale(geo3)+ scale(geo4)+ scale(geo5)+ scale(geo6), ~ scale(latitude), ~BasinType |
| without geo2 | -129.0061 | 0.857 | 0.273 | 0 | gen_dist, ~scale(geo1)+ scale(geo3)+ scale(geo4)+ scale(geo5)+ scale(geo6), ~ scale(latitude), ~BasinType |
| without latitude | -129.9892 | 0.835 | 0.215 | 0 | gen_dist, ~scale(geo1)+ scale(geo3)+ scale(geo4)+ scale(geo5)+ scale(geo6), ~BasinType |
| without geo6 | -131.1035 | 0.803 | 0.139 | 0 | gen_dist, ~scale(geo1)+ scale(geo3)+ scale(geo4)+ scale(geo5), ~BasinType |

| | | | | | |
|---|---|---|---|---|---|
| without geo4 | -132.1262 | 0.788 | 0.099 | geo1 | gen_dist, ~scale(geo1)+ scale(geo3)+ scale(geo5), ~BasinType |
| without geo5 | -132.4334 | 0.799 | 0.1 | 0 | gen_dist, ~scale(geo1)+ scale(geo3), ~BasinType |
| **without latitude** | -130.3606 | 0.789 | 0.239 | 0 | gen_dist, ~ scale(geo1)+ scale(geo2)+ scale(geo3)+ scale(geo4)+ scale(geo5)+ scale(geo6), ~ scale(Altitude), ~BasinType + system |
| from here we start to extract variables from the model without latitude (we remove those with the highest p-value) | | | | | |
| without system | -132.2147 | 0.715 | 0.127 | altitud and basin | gen_dist, ~ scale(geo1)+ scale(geo2)+ scale(geo3)+ scale(geo4)+ scale(geo5)+ scale(geo6), ~ scale(Altitude), ~BasinType |
| without geo6 | -133.0721 | 0.686 | 0.071 | altitud and basin | gen_dist, ~scale(geo1)+ scale(geo2)+ scale(geo3)+ scale(geo4)+ scale(geo5),~ scale(Altitude), ~BasinType |
| without geo1 | -134.1869 | 0.659 | 0.05 | altitud and basin | gen_dist, ~scale(geo2)+ scale(geo3)+ scale(geo4)+ scale(geo5),~ scale(Altitude), ~BasinType |
| without geo2 | -134.9452 | 0.646 | 0.022 | altitud and basin | gen_dist, ~scale(geo2)+ scale(geo3)+ scale(geo4),~ scale(Altitude), ~BasinType |
| without geo5 | -135.8216 | 0.626 | 0.015 | altitud and basin | gen_dist, ~ scale(geo3) + scale(geo4), ~ scale(Altitude), ~BasinType |
| without geo4 | -136.0251 | 0.6486 | 0.01 | altitud and basin | gen_dist, ~ scale(geo3), ~ scale(Altitude), ~BasinType |
| without geo3 | -135.2652 | 0.729 | 0.013 | altitud and basin | gen_dist, ~ scale(Altitude) + BasinType |

* geo 1 to 6 are the axes given by the PCNM representing the geographical distance (IBD)
